# Global Patterns and Drivers of Climatic Niche Originality and Overlap: Disentangling Ecological From Methodological Signals

**DOI:** 10.1002/ece3.74215

**Published:** 2026-09-03

**Authors:** Mathieu Chevalier, Clement Violet, Aurélien Boyé

**Affiliations:** ^1^ Centre de Bretagne, DYNECO, Laboratoire d'Ecologie Benthique Côtière (LEBCO) IFREMER Plouzané France; ^2^ ISEM Univ Montpellier, CNRS, EPHE, IRD Montpellier France

**Keywords:** geographic range overlap, macroecology, niche originality, niche overlap, null expectations, null models, phylogenetic isolation, species co‐occurrence

## Abstract

Niche overlap is widely used to study species coexistence and biodiversity patterns. However, because macroclimatic niches are typically estimated from species distributions, relationships between niche overlap and geographic range overlap may partly reflect the shared structure of environmental and geographic space rather than ecological processes. Here, we address this challenge by constructing the BioOverlap database combining IUCN range maps and bioclimatic variables to quantify macroclimatic niche properties, niche overlap, and geographic range overlap among ~16,000 terrestrial and marine species, yielding ~90 million pairwise comparisons. Using a taxonomically structured subset encompassing eight taxonomic groups with consistently available variables, we addressed two objectives. First, we quantified niche originality (i.e., the distinctiveness of a species' niche relative to other species), and identified its ecological and evolutionary determinants. Second, we used structural equation modeling integrating niche and range attributes to test whether relationships between niche and range overlap among sympatric species deviate from null expectations. Niche originality showed consistent associations with ecological variables including niche breadth, climatic heterogeneity, species richness and productivity, whereas phylogenetic isolation had limited influence. In contrast, across most sympatric taxa, the positive association between niche and range overlap was indistinguishable from null expectations, with deviations observed only in birds, Chondrichthyes, and marine fishes. This suggests that relationships between niche and range overlap estimated from distribution data may arise largely from the shared structure of species distributions and environmental space rather than from ecological structuring. Explicit null model comparisons are therefore essential when interpreting niche–range relationships and suggest that independently estimated niches (e.g., from experimental data) may be necessary to distinguish genuine ecological signal. Together, these results establish BioOverlap as a valuable global resource for investigating large‐scale biodiversity patterns and niche structure across taxa, while providing a framework for benchmarking niche‐based relationships against appropriate null expectations.

## Graphical abstract

This study presents BioOverlap, a global database quantifying macroclimatic niche properties, niche overlap, and geographic range overlap for ~16,000 species. Analyses reveal that niche originality consistently reflects ecological factors such as niche breadth, productivity, and climatic heterogeneity, whereas pairwise niche–range relationships are largely consistent with null expectations, except in birds, Chondrichthyes, and marine fishes. These results highlight the importance of null models in distinguishing genuine ecological signals from geometric and statistical constraints in large‐scale niche analyses.

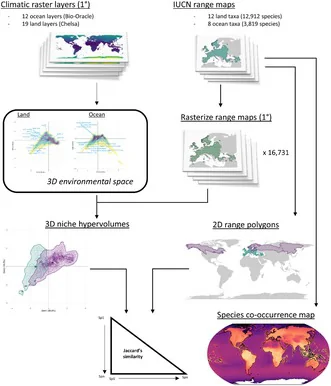

## 1 Introduction

An overarching goal of ecology is to understand the drivers that promote and maintain biodiversity (Hutchinson 1957; Kerner 1974). Niche theory has been central to this endeavor (Worm and Tittensor 2018), providing a conceptual framework in which species are viewed as occupying an n‐dimensional hypervolume defined by the set of environmental conditions under which they can persist and beyond which fitness dramatically decreases (Hutchinson 1957; Peterson et al. 2011). Within this framework, the realized niche (i.e., the subset of conditions where species are actually observed) emerges from the interplay between abiotic constraints, biotic interactions and dispersal limitations (Jackson and Overpeck 2000; Peterson et al. 2011). This framework has supported the development of a wide range of ecological and evolutionary hypotheses linking species' environmental requirements to patterns of diversity, distribution, and coexistence (Chase and Leibold 2003; Godsoe et al. 2017; Worm and Tittensor 2018). Consequently, quantifying ecological similarity among species has become fundamental to understanding biodiversity patterns across ecological disciplines, from community assembly and biogeography to conservation and global change ecology (Box 1).

BOX 1 **Potential applications of the BioOverlap database.**

The BioOverlap database provides standardized estimates of climatic niche and geographic range characteristics, along with measures of niche overlap and geographic range overlap for more than 16,000 terrestrial and marine species. Because these metrics are calculated consistently across taxonomic groups, the database offers opportunities to address a broad range of questions spanning ecology, evolution, biogeography, and conservation. Whereas niche overlap characterizes ecological similarity between pairs of species, niche originality quantifies the relative isolation of individual species in environmental space, allowing researchers to identify environmentally distinctive taxa and assess their contribution to ecological diversity (Pavoine et al. 2017; Pavoine and Ricotta 2021).

In community ecology, BioOverlap can be used to investigate how environmental similarity influences species coexistence, community assembly and patterns of biodiversity turnover across spatial scales. Pairwise niche overlap metrics may help evaluate the relative roles of environmental filtering, niche differentiation, and historical contingency in shaping species assemblages (Chesson 2000; Barabás et al. 2018). Species‐level niche originality can also provide complementary information by identifying environmentally unique species that may contribute disproportionately to functional or ecological differentiation within communities (Violle et al. 2017).

In macroecology and biogeography, the database enables analyses of how patterns in environmental space relate to species distributions, geographic co‐occurrence, and large‐scale biodiversity gradients. Combined with null‐model approaches, these metrics can help disentangle ecological processes from patterns emerging through the geometry of species ranges and environmental space (Colwell and Rangel 2009; Coelho et al. 2023; Graham et al. 2024). Patterns of niche originality can also reveal how ecological uniqueness is distributed across regions, helping identify environmentally distinctive taxa and gradients of specialization (Gouveia et al. 2014).

In evolutionary ecology, BioOverlap can be used to investigate niche conservatism, niche divergence, and the relationship between evolutionary history and environmental specialization. Integrating niche overlap metrics with phylogenetic information may provide insights into the extent to which ecological similarity is maintained or modified through evolutionary time (Losos 2008; Wiens et al. 2010). Niche originality can also be used to investigate whether evolutionarily isolated or rapidly diverging lineages occupy environmentally distinctive regions of niche space, providing a complementary perspective on ecological diversification (Tucker et al. 2017; Kondratyeva et al. 2019; Pavoine and Ricotta 2021).

In conservation biology, the database can support assessments of ecological redundancy, environmental distinctiveness, and species vulnerability. Niche originality metrics can identify species occupying rare or isolated portions of environmental space that may represent irreplaceable components of biodiversity. Combined with measures of ecological redundancy, extinction risk, and climate‐change projections, originality can help prioritize species whose loss would disproportionately reduce the diversity of ecological strategies represented within regional or global biotas (Pavoine et al. 2017; Carmona et al. 2021).

The database may also facilitate studies of biological invasions, species redistributions, and emerging ecological interactions under global change. Quantifying environmental similarity among native and introduced taxa can help evaluate establishment potential, niche shifts, and the emergence of novel species associations in rapidly changing ecosystems (Pearman et al. 2008). Changes in niche originality through time may also reveal whether species are becoming increasingly ecologically unique or redundant under climate change and biological invasions (Bates and Bertelsmeier 2021).

BioOverlap also contains overlap estimates among species belonging to different taxonomic groups. This feature opens opportunities for investigating cross‐taxon patterns of environmental similarity, potential interaction networks, trophic relationships, host–parasite associations, and the degree of niche convergence among evolutionarily distant lineages (Pigot et al. 2018; Carroll et al. 2019; Suraci et al. 2022). Such analyses may help identify ecological links that are not apparent from geographic distributions alone.

More broadly, BioOverlap provides a standardized framework for comparative analyses of niche structure and species overlap across taxa and ecosystems. Its consistent methodology facilitates the integration of environmental, geographic, and evolutionary information, enabling researchers to address questions that extend beyond the applications explored in the present study.

Many of these applications will benefit from integration with complementary data sources, including functional traits, physiological tolerances, trophic information, phylogenetic data, species interactions, or demographic observations, to distinguish ecological mechanisms from patterns arising through shared environmental or geographic structure.

A central metric for assessing species similarity is niche overlap, which quantifies the degree of similarity between species along one or several ecological dimensions (Geange et al. 2011; Broennimann et al. 2012). Niche overlap can be quantified using different descriptors of species ecology, including climatic conditions (Grinnellian niche; Austin 2002), trophic resource use (Eltonian niche; Leibold 1995) or functional traits that capture ecological strategies and responses to environmental gradients (Violle et al. 2017), and can be measured between species, across time periods, or between geographic regions. It has been widely used to infer ecological processes such as competition (Pianka 1974; Godwin et al. 2020), trophic interactions (Carroll et al. 2019; Suraci et al. 2022), invasion dynamics (Pearman et al. 2008) or community assembly mechanisms (Chesson 2000; Barabás et al. 2018). At broad spatial scales, relationships between niche overlap and geographic range overlap have often been interpreted as evidence that shared environmental requirements constrain species distributions and promote co‐occurrence (Davies et al. 2007; Pigot et al. 2018). Although niche overlap is one of the most widely used descriptors of ecological similarity, it does not capture how individual species are positioned within the broader environmental space. Complementary species‐level metrics therefore seek to characterize ecological distinctiveness relative to the rest of the species pool. For instance, niche originality (sometimes referred to as marginality or ecological distinctiveness) quantifies how isolated a species is in environmental space relative to other species (sensu the originality framework developed in functional and phylogenetic ecology; see Pavoine et al. 2017; Kondratyeva et al. 2019; Pavoine and Ricotta 2021). Such approaches have been applied across spatial scales, from local assemblages to macroecological studies (Gouveia et al. 2014; Violle et al. 2014). Together, niche overlap and niche originality provide complementary perspectives on niche structure by quantifying, respectively, pairwise ecological similarity and the position of individual species within environmental space.

Niche overlap and niche originality have been applied across a wide range of research fields, including population dynamics, trophic ecology, invasion biology, community ecology, and conservation (Box 1), making them key tools for linking ecological theory with large‐scale biodiversity patterns and for predicting species responses to global change (Bates and Bertelsmeier 2021). Different strategies have been developed to quantify these metrics, ranging from univariate approaches focusing on individual niche axes (Mason et al. 2011; Waldock et al. 2019) to multivariate methods that estimate overlap and species positions within high‐dimensional environmental spaces (Swanson et al. 2015; Carvalho and Cardoso 2020). Although niche similarity can be characterized using diverse ecological information, such as isotopic data to characterize trophic niches (Lesser et al. 2020) or trait data to describe ecological strategies (Violle et al. 2007), these datasets often remain taxonomically or geographically incomplete, limiting their application at broad spatial scales (Tyler et al. 2012; Hortal et al. 2015). In contrast, climatic data are globally available and can be combined with species distribution records to estimate macroclimatic niches across a broad range of taxa. Because climate is a major abiotic determinant of species distributions and physiological performance (Rödder and Engler 2011; Broennimann et al. 2012), climatic niche metrics derived from distribution data have become the dominant approach for large‐scale comparative analyses. The increasing availability of global databases, such as IUCN range maps and high‐resolution climatic datasets, now provides unprecedented opportunities to quantify niche similarity across thousands of species (Trisos et al. 2020; Carmona et al. 2021). Yet, this widespread empirical framework introduces an important conceptual challenge: while theory and ecological inference are generally framed in terms of species' fundamental niches, in practice, these niches are usually estimated from observations collected in geographic space (Matthiopoulos 2022).

This dependence between environmental and geographic representations of biodiversity is formalized by Hutchinson's duality, which describes the reciprocal mapping between environmental and geographic space (Hutchinson 1957; Colwell and Rangel 2009). In principle, any environmental condition corresponds to one or more geographic locations, whereas each geographic location at a given time occupies a unique position in environmental space, allowing information to be translated between the two. In practice, environmental conditions are rarely observed independently of geographic space because they are typically extracted from species' recorded distributions using gridded climatic datasets (Broennimann et al. 2012; Graham et al. 2024). As a result, environmental similarity and geographic co‐occurrence are intrinsically linked: species that overlap geographically are likely to share at least part of their environmental space, whereas species occupying similar environments do not need to co‐occur geographically. Recent studies have shown that biodiversity patterns can differ markedly depending on whether they are analyzed in environmental or geographic space, underscoring that these two representations are complementary but not interchangeable (Coelho et al. 2023; Graham et al. 2024). Recognizing this dependence is therefore a prerequisite for determining whether relationships between niche similarity and geographic overlap reflect ecological processes or simply the way environmental niches are estimated from distribution data.

The consequences of this dependence become increasingly pronounced when niche theory is scaled from local communities to regional and global biodiversity patterns (Vellend 2010; HilleRisLambers et al. 2012). Species distributions and environmental niches are inherently scale dependent, and the correspondence between environmental and geographic space can change across spatial grains and extents (Peterson et al. 2011; Mertes and Jetz 2018; Lu and Jetz 2023). At broad scales, biodiversity patterns emerge not only from the characteristics of individual species but also from how species distributions overlap across space. Consistent with this view, diversity‐fields approaches describe patterns of species richness, coexistence, and range overlap as emergent properties of the spatial arrangement of species ranges (Arita et al. 2008; Villalobos et al. 2013), highlighting pairwise overlap as a fundamental component of large‐scale biodiversity patterns.

Interpreting this pattern, however, is far from straightforward. At local scales, niche differences can be directly linked to coexistence through mechanisms such as resource partitioning and stabilizing interactions (Chesson 2000; Chase and Leibold 2003). In contrast, regional and global patterns of co‐occurrence emerge from the combined effects of environmental filtering, dispersal limitation, historical contingency, and stochastic processes (Ricklefs 1987; Vellend 2016). Consequently, relationships between environmental similarity and geographic overlap may arise even when local ecological interactions are weak or absent (Soberón 2007). Conversely, species with similar environmental niches may remain geographically separated due to dispersal barriers, historical factors, or biogeographic constraints (Peterson et al. 2011; Guisan et al. 2017). As the relationship between environmental similarity and geographic overlap becomes increasingly indirect across scales, observed niche‐range relationships cannot be interpreted as direct evidence of ecological processes, making it essential to distinguish genuine ecological signals from patterns generated by spatial structure, stochastic processes, or methodological constraints.

These conceptual challenges highlight the need to evaluate niche‐based relationships against appropriate null expectations rather than interpreting observed patterns in isolation. Null‐model comparisons provide a baseline expectation of niche‐range relationships under random assembly, making it possible to determine whether observed correlations exceed patterns expected from the structure of species distributions alone (Gotelli and Graves 1996). Without such benchmarks, macroecological studies risk overinterpreting relationships that primarily reflect the shared data structure, range geometry, or the distribution of environmental conditions across geographic space rather than ecological processes (Colwell and Lees 2000; Raes and Steege 2007; Peterson and Soberón 2012). A different inferential issue arises when considering species‐level metrics such as niche originality. Rather than resulting from nonindependence between environmental and geographic representations, apparent differences in originality may be influenced by species' geographic range size, because widespread species often occupy broader portions of environmental space and may therefore appear less environmentally distinct simply due to their distributional extent (Slatyer et al. 2013). Distinguishing these inferential contexts (i.e., structural dependence in niche‐range overlap versus potential confounding in species‐level originality) is critical for assessing when large‐scale niche metrics provide robust ecological insight. Yet, despite their importance, null‐model approaches remain underused in cross‐taxon analyses, limiting our ability to separate ecological signal from methodological constraints and stochastic expectations (Weiher and Keddy 1995; Münkemüller et al. 2012).

Despite the widespread use of niche‐based metrics in ecology and biogeography, we still lack a comprehensive understanding of how species‐level and pairwise niche metrics vary across major taxonomic groups and whether commonly reported niche‐range relationships exceed expectations generated by null models. A global assessment of niche originality, niche overlap, and geographic range overlap across taxonomic groups is therefore needed to determine which patterns are consistent at broad scales, and under which conditions niche‐based metrics provide meaningful ecological insight. Here, we address this challenge by combining IUCN range maps with global climatic data to construct the BioOverlap database, encompassing more than 16,000 terrestrial and marine species. Using a subset of this dataset, we first examined the determinants of niche originality across taxa to identify the environmental, geographic, and phylogenetic factors associated with species positions in environmental space at global scales. We then investigated the drivers of species coexistence using a structural equation modeling framework integrating niche and range attributes along with phylogenetic information, and tested whether relationships between niche overlap and geographic range overlap deviate from null expectations (Gotelli and Graves 1996). By comparing observed patterns with null expectations across multiple taxonomic groups, we aim to determine the extent to which commonly reported niche‐range relationships reflect ecological structuring rather than stochastic processes, spatial constraints, or methodological artifacts. Finally, we illustrate how the BioOverlap database can support a broad range of ecological and evolutionary applications (Box 1), while providing guidance on when niche‐based metrics are informative and when they should be interpreted with caution.

## 2 Methods

### 2.1 Datasets

#### 2.1.1 Data to Build BioOverlap

##### 2.1.1.1 Species Geographical Ranges

We obtained range maps for 54,147 species from the International Union for Conservation of Nature (IUCN) Red List, covering 20 major taxonomic groups including terrestrial vertebrates, plants, marine fishes, invertebrates, and foundational marine species such as corals and seagrasses. To ensure biological relevance and consistency across taxa, we retained only polygons labeled as “extant,” “native,” “reintroduced,” or “assisted colonization.” For terrestrial species, analyses were restricted to breeding ranges, while marine species confined to depths below 200 m were excluded to focus on the epipelagic zone where environmental variation is most directly experienced.

##### 2.1.1.2 Climatic Data to Define Species Niches

To characterize terrestrial climates, we extracted 19 bioclimatic variables from the CHELSA V2 dataset (https://chelsa‐climate.org/; Karger et al. 2017). These variables include measures of central tendency (e.g., annual mean temperature), variability (e.g., temperature and precipitation seasonality), and climatic extremes (e.g., minimum temperature of the coldest month, precipitation of the driest quarter), all of which are known to influence species' physiological tolerances and distribution limits. For marine systems, we extracted 12 environmental layers from the Bio‐ORACLE V2 database (https://www.bio‐oracle.org/; Tyberghein et al. 2012; Assis et al. 2018), including minimum, maximum, mean, and range values for sea surface temperature, salinity, and current velocity within the upper 200 m. These variables represent key abiotic drivers of marine species distributions, influencing metabolism, dispersal, and habitat suitability.

All climatic variables were aggregated to a common spatial resolution of 1° to ensure consistency with species distribution data across terrestrial and marine realms.

#### 2.1.2 Data for Modeling Niche Originality and Co‐Occurrence Patterns

##### 2.1.2.1 Environmental Predictors

To explain patterns in niche originality, we compiled spatial predictors capturing key environmental dimensions known to influence species distributions and ecosystem functioning. Climatic rarity and climatic heterogeneity were derived following Fournier et al. (2020). Climatic rarity, calculated using the climate frequency index, quantifies the spatial frequency of climatic conditions, with low values indicating rare climates and high values common ones. Climatic heterogeneity, calculated using the climate heterogeneity index, measures the spatial variability of climatic conditions within a grid cell or region and reflects environmental complexity, which can promote niche differentiation and species coexistence.

We additionally quantified species richness to assess how niche properties vary along gradients of species richness. For this purpose, we rasterized species ranges at a 1° spatial resolution (~10,000 km^2^ at the equator), providing a compromise between computational feasibility and ecological realism, and reducing potential biases associated with overestimation of presences. Although this resolution is too coarse to capture fine‐scale habitat or resource preferences, it is appropriate for characterizing macroclimatic niches at the global scale (Hurlbert and Jetz 2007), where climate acts as a primary abiotic filter structuring species distributions (Worm and Tittensor 2018). We then stacked these rasterized species range maps within each taxonomic group, generating spatially explicit layers of local diversity.

Finally, we included net primary productivity from Zhao et al. (2005) as a proxy for ecosystem productivity and energy availability. Net primary productivity represents the balance between carbon assimilation through photosynthesis and plant respiration, and is a key determinant of biomass accumulation, trophic structure, and large‐scale patterns of species diversity.

##### 2.1.2.2 Phylogenetic Data

Phylogenies for birds, amphibians, reptiles, mammals, ray‐finned fishes, cartilaginous fishes, and plants were constructed using integrative frameworks that combine molecular data, taxonomic information, and fossil calibrations to generate large, time‐calibrated trees. The global bird tree (Jetz et al. 2012) was built using Bayesian relaxed‐clock models across 158 clades, with unsampled species placed via taxonomic constraints and birth–death models. Amphibian and reptile trees (Tonini et al. 2016; Jetz and Pyron 2018) employed multilocus backbones, probabilistic placement of missing taxa, and relaxed‐clock dating to generate posterior distributions of 10,000 trees from which a consensus tree was derived. The mammal phylogeny (Upham et al. 2019) integrated a 31‐gene supermatrix with taxonomic imputation for missing species and fossil‐based calibration of backbone and patch clades. The ray‐finned fish tree (Rabosky et al. 2018) used a curated 27‐gene supermatrix and maximum‐likelihood inference, supported by taxonomic reconciliation. For Chondrichthyes (Stein et al. 2018), a 15‐locus supermatrix was assembled from public and novel sequence data, analyzed using RAxML and treePL for topology and divergence dating. Rogue taxa were filtered, and unsampled species were added using taxonomic rules and BEAST‐based polytomy resolution, resulting in 10,000 fully resolved, dated trees. Plant phylogenies were based on the PhytoPhylo megaphylogeny (Qian and Jin 2016), an updated version of Zanne et al. (2014) with over 2000 taxonomic corrections and time calibration. The S.PhyloMaker R function was used to place missing taxa under alternative scenarios, enabling robust phylogenies even in the absence of genetic data.

All phylogenies were pruned to include only species for which overlap data were available (see below). Species with computed overlap but absent from the phylogeny were excluded from subsequent analyses. For each species pair, we calculated cophenetic distances as a measure of evolutionary divergence, where higher values indicate greater phylogenetic separation. To characterize species‐level phylogenetic isolation, we calculated, for each species, the minimum cophenetic distance to any other species in the phylogeny. Higher values indicate species that are more isolated from their closest relative in the dataset. This metric (termed *phylogenetic isolation* hereafter) captures nearest‐neighbor phylogenetic isolation and should not be interpreted as a general measure of phylogenetic rarity or evolutionary distinctiveness (Tucker et al. 2017; Pavoine and Ricotta 2021).

### 2.2 Analytical Framework

#### 2.2.1 Construction of the BioOverlap Database

Using species ranges and climatic data, we constructed the BioOverlap database by first estimating species' climatic niches and then quantifying geographic and niche overlap among all species pairs.

##### 2.2.1.1 Estimating Climatic Niches

We reduced the dimensionality of the climate data separately for each realm (marine and terrestrial) via two principal component analyses (PCA using the ade4 R package; Dray and Dufour 2007) performed on the 19 and 12 bioclimatic variables presented above. From each analysis, we retained the first three components which together explained 83.9% of the total variation in terrestrial climates and 78.7% in marine climates. Species' macroclimatic niches were then defined as hypervolumes in this three‐dimensional PCA space, using a Gaussian kernel density estimation (KDE) algorithm implemented in the hypervolume R package (Blonder 2019). To ensure the robustness of the KDE used to compute species climatic hypervolumes, we followed established best practices (Blonder et al. 2014). Climatic variables were scaled and centered prior to constructing the PCA space to standardize units and reduce variance‐related biases. We included only species with 30 unique presence points in environmental space to avoid unreliable estimates due to undersampling (Blonder et al. 2014). Although strict multivariate normality is not a requirement for KDE, we confirmed for a subset of species that the point distributions in environmental space produced smooth and interpretable hypervolumes, consistent with KDE assumptions. After removing rare species (i.e., those with less than 30 points) we obtained a final dataset of 16,731 species (12,912 terrestrial and 3819 marine).

##### 2.2.1.2 Quantifying Range and Niche Overlap

We quantified both geographic and environmental similarity for all species pairs within each realm to explore patterns of regional co‐occurrence and niche overlap. Regional co‐occurrence (i.e., geographic overlap) was estimated based on species' range maps (i.e., 2D polygons). We computed pairwise range overlap by assessing the spatial intersection of these polygons in geographic space. Specifically, we calculated the Jaccard and Sørensen indices, which measure the proportion of shared range area relative to the union or average area of the two species' ranges, respectively. We also computed the area of each species' range (termed *range size* hereafter), the Euclidean distance between range centroids, and the minimum great‐circle distance (km) separating the boundaries of two species distributions, irrespective of direction to assess both overlap and spatial proximity. When ranges overlap, the distance separating ranges is equal to zero.

Pairwise niche overlap was quantified as the intersection volume between the 3D hypervolumes projected within the PCA space using the hypervolume R package (Blonder 2019). As with range overlap, we calculated Jaccard and Sørensen indices to express niche similarity in relative terms. Additionally, we extracted the volume of each species' hypervolume (termed *niche breadth* hereafter), the Euclidean distance between hypervolume centroids, and the minimum edge‐to‐edge distance between hypervolumes to capture both overlap and separation in niche space.

All pairwise similarity metrics, along with species‐level descriptors such as niche breadth and range size, were compiled into a structured relational database, termed BioOverlap (Chevalier et al. 2024), designed to support large‐scale comparative analyses of niche overlap, regional co‐occurrence, niche structure and biodiversity patterns, and to evaluate their interpretation in light of null expectations (see Box 1). Because Jaccard and Sørensen indices are monotonically related and provide largely redundant information (Legendre and Legendre 2012), we report both metrics in the BioOverlap database to maximize flexibility for future users. However, to avoid redundancy in our empirical analyses, all subsequent analyses were conducted using the Jaccard index.

**FIGURE 1:**
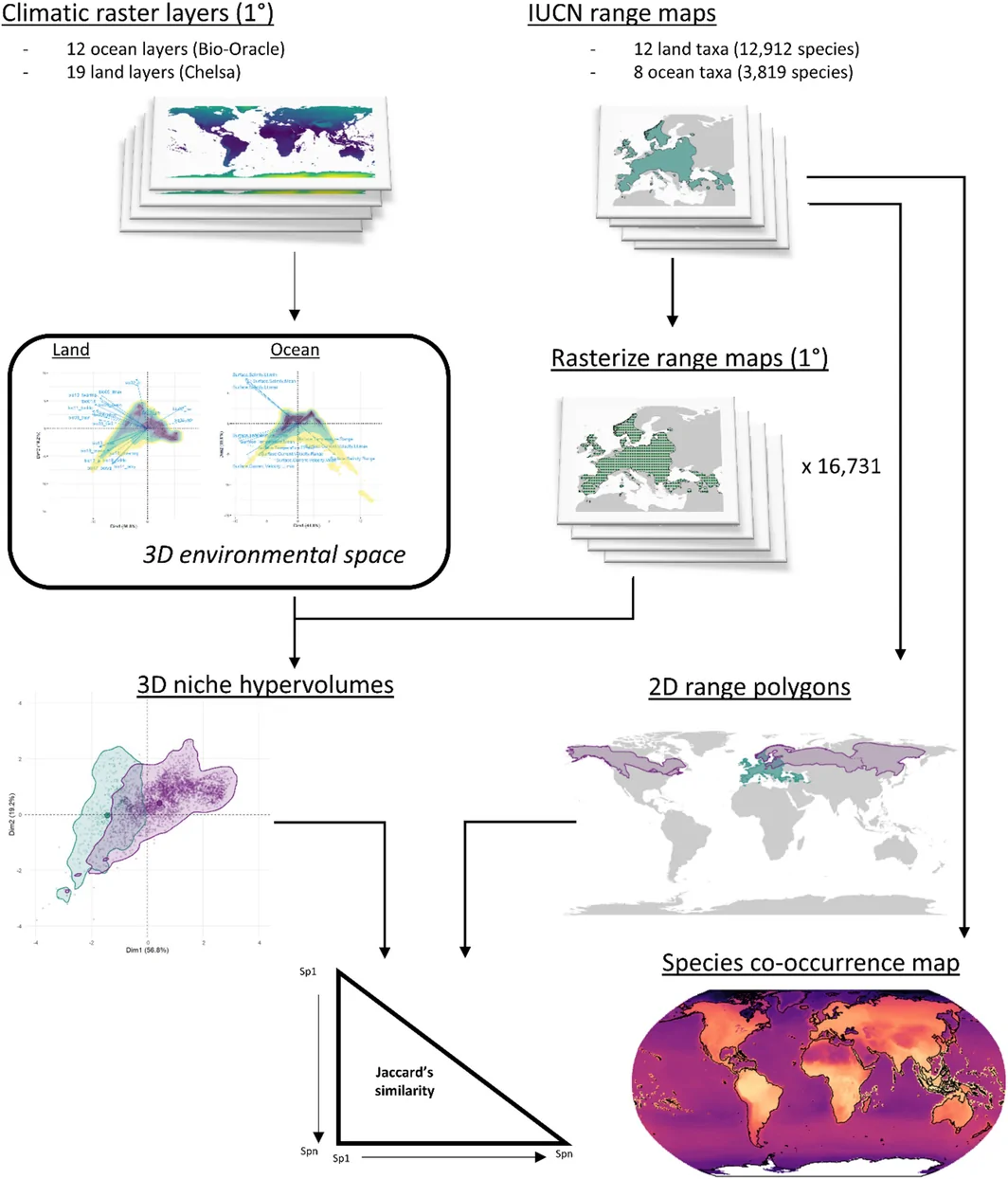
Modeling workflow illustrating the integration of IUCN range maps and climatic layers to estimate niche and range overlap between species pairs (reproduced with permission from Chevalier et al. 2024). Species' geographic ranges (from IUCN) are overlaid with climatic data to extract environmental conditions across their distributions. These conditions are then used to construct niche hypervolumes in climate space for each species. For visualization purposes, the hypervolumes are shown in two dimensions, but all analyses were conducted using three‐dimensional environmental space. Niche overlap is calculated as the intersection of these hypervolumes, while range overlap is derived from the spatial intersection of species' geographic ranges. Intersections were calculated using Jaccard and Sorensen indices.

The workflow describing the combination of IUCN range maps with climatic data to obtain measures of range and niche overlap is shown in Figure 1 (see Chevalier et al. 2024).

#### 2.2.2 Modeling Niche Originality and Co‐Occurrence Patterns

In the following analyses, we focused on eight taxonomic groups (amphibians, birds, Chondrichthyes, freshwater and marine fishes, terrestrial mammals, plants and reptiles) for which we had information on phylogeny and at least 30 pairwise measures of overlap. This resulted in the selection of 10,823 species and over 2 million species pairs.

##### 2.2.2.1 Identifying Ecologically Distinct Species and Associated Drivers

To quantify species' niche originality, we first constructed pairwise dissimilarity matrices for each taxonomic group using values of 1—niche overlap as a measure of ecological dissimilarity between species. We then performed a principal coordinate analysis (PCoA), a distance‐based ordination method also known as classical multidimensional scaling, on each dissimilarity matrix to project species into a continuous, multidimensional niche dissimilarity space (Legendre and Legendre 2012). Within this space, we followed the framework developed by Violle et al. (2017) and calculated, for each species, the Euclidean distance to the nearest neighbor, which provides a quantitative measure of how isolated or distinct a species is in terms of its climatic niche (termed *niche originality*). Species with higher distances were considered to have more original niches, reflecting greater ecological distinctiveness within environmental space. To check the sensitivity of this analysis to outlier effects, we also measured niche originality considering the average distance to the 10 closest neighbors.

We modeled species' niche originality as a function of ecological, climatic, and evolutionary variables. These included range‐level predictors such as climatic heterogeneity, climatic rarity, net primary productivity and species richness along with species‐level attributes such as phylogenetic isolation, niche breadth and range size. These predictors were selected because they represent factors potentially associated with ecological distinctiveness (Slatyer et al. 2013; Violle et al. 2017). For instance, climatic heterogeneity may have contrasting effects on niche originality. On one hand, species inhabiting heterogeneous environments may exhibit broader niches and overlap with more specialized taxa, resulting in lower originality. On the other hand, environmental complexity may promote niche differentiation, leading to higher originality (Enquist et al. 2019). Similarly, species occurring in climatically rare environments are expected to exhibit specialized adaptations, often associated with narrower niche breadth and smaller range size, and thus higher niche originality (Fournier et al. 2020). From these hypotheses, it follows that climatic effects are partially constrained by other factors with broader niches and larger ranges generally associated with more generalized ecological strategies (Colwell and Futuyama 1971). High productivity can support a greater number of species with similar niches, potentially reducing niche originality (Ricklefs 2012). Conversely, reduced resource limitation in productive environments may facilitate finer niche partitioning and increased ecological differentiation (Evans et al. 2005). Likewise, high local species richness may either constrain originality through increased competition or promote it via niche divergence and character displacement (Macarthur and Levins 1967a). Finally, phylogenetic isolation may reflect evolutionary divergence associated with ecological distinctiveness, particularly when key functional traits are phylogenetically conserved (Losos 2008).

For each species, range‐level predictors were obtained by overlaying its IUCN range polygon onto the corresponding global raster layers and averaging values across its geographic range. All predictors were standardized to *z*‐scores (mean = 0, variance = 1) prior to inclusion in linear models to allow comparing their effect sizes and therefore their importance in driving niche originality. We only considered linear additive effects for simplicity but acknowledge that interactions and nonlinear effects can also be possible. Separate models were fitted for each taxonomic group, and the contribution of each predictor was quantified using partial *R* ^2^ values derived from Type II sums of squares, representing the proportion of variance uniquely explained by each variable after accounting for the effects of the other predictors in the model. All models met standard assumptions of normality, collinearity, and homoscedasticity, ensuring reliable inference across diverse ecological contexts.

##### 2.2.2.2 Evaluating the Drivers of Range Overlap and Deviations From Null Expectations

To investigate the factors associated with regional coexistence, we modeled range overlap between species pairs as a function of ecological and phylogenetic variables. Because both niche overlap and range overlap are derived from the same occurrence data, these metrics are inherently prone to statistical coupling. Therefore, we explicitly evaluated observed relationships against null expectations.

We applied saturated structural equation models (SEMs; Grace 2006) separately for each taxonomic group. The SEM was constructed from an a priori causal framework represented by a directed acyclic graph (DAG; Figure 4A). The DAG formalizes our hypothesis that geographic overlap among species pairs is influenced both directly and indirectly through niche overlap. Specifically, phylogenetic distance, geographic distance between ranges, range size, climatic niche breadth, and environmental heterogeneity were hypothesized to influence niche overlap and/or geographic overlap. Because our observations were dyadic, range size and climatic niche breadth could not be represented as species‐specific predictors in the pairwise models. We therefore characterized these attributes using the maximum value within each species pair. This choice reflects our expectation that pairwise overlap would be influenced primarily by the species with the largest range or broadest niche, which sets the greater potential extent of geographic or environmental overlap. Niche overlap was expected to mediate part of the effects of these variables on geographic overlap. The SEM comprised three component models estimating (i) predictors of niche overlap, (ii) predictors of geographic overlap, including the indirect pathway mediated through niche overlap, and (iii) predictors of maximum niche breadth (Figure 4A). Together, these models quantify the relative contributions of environmental similarity and other range‐related factors to geographic overlap while accounting for repeated observations involving the same species through crossed random effects.

Path directions were specified a priori based on ecological theory and previous work on Hutchinson's duality and the relationship between environmental and geographic space (Colwell and Rangel 2009; Coelho et al. 2023; Graham et al. 2024). Models were fitted using the piecewiseSEM R package (Lefcheck 2015) with linear mixed‐effects models. Because observations corresponded to species pairs rather than individual species, conventional comparative methods based on a single phylogenetic covariance matrix (e.g., phylogenetic generalized least square models) were not directly applicable. Instead, phylogenetic distance between species was included explicitly as a predictor, while species identity was incorporated as a random effect to account for repeated participation of species across multiple pairs. Only species pairs with nonzero range overlap were included, reflecting sympatric interactions. The piecewise SEM were specified as saturated models, implying that all hypothesized causal pathways were included in the network. Consequently, the models implied no conditional independence claims, resulting in zero degrees of freedom and rendering global goodness‐of‐fit statistics (Fisher's C and the associated chi‐square test) uninformative. Model evaluation therefore focused on the explanatory power of individual component models, quantified using marginal and conditional *R* ^2^ values. Marginal *R* ^2^ represents the variance explained by fixed effects alone, whereas conditional *R* ^2^ represents the variance explained by both fixed and random effects. Visual inspection of the diagnostic plots did not reveal any major violations of model assumptions or obvious lack of fit (Text S1, Figures S1–S8, Table S1).

To account for potential methodological bias arising from statistical dependence between niche and range attributes, we implemented 100 null model simulations for each taxonomic group (Gotelli and Graves 1996). For each of null model, we generated random assemblages of 500 species, representing species ranges as circular polygons of varying sizes. Ranges were placed randomly within the terrestrial realm for terrestrial taxa (e.g., amphibians, mammals, birds, and plants) or within the marine realm for marine taxa (e.g., marine fish, Chondrichthyes), independent of environmental conditions. From these simulated ranges, species' environmental niches were inferred, and niche overlap and range overlap were computed for all species pairs using the same procedures as for the observed data. Because simulated species do not correspond to real taxa, phylogenetic distances were not included in the null SEMs. Incorporating arbitrary or randomly pruned phylogenies would impose an artificial covariance structure unrelated to the simulated ecological variables, thereby confounding interpretation of null expectations (Webb et al. 2002; Revell 2012). Null SEMs therefore included only ecological variables (niche overlap, centroid distance, maximum range size, and maximum niche breadth), producing distributions of SEM path coefficients expected under no ecological or evolutionary structure (Lefcheck 2015). Observed SEM coefficients were then compared to these null distributions to compute standardized effect sizes (SES) and empirical *p*‐values, quantifying the degree to which observed relationships exceeded random expectations (Garrido et al. 2022). Standardized effect sizes (SES) were calculated as:

$$\mathrm{SES}=\frac{\beta_{\mathrm{obs}}–\text{mean}\left(\beta_{\text{null}}\right)}{\mathrm{SD}\left(\beta_{\text{null}}\right)}$$

where β_obs_ is the observed path standardized coefficient and β_null_ corresponds to the distribution of standardized coefficients obtained from null simulations. SES therefore quantify the extent to which observed relationships deviate from null expectations expressed in units of null‐model standard deviations. Together, this combination of observed SEMs and null‐model comparisons enables a comprehensive evaluation of the multivariate relationships among niche overlap, range overlap, and associated predictors while explicitly controlling for potential statistical artifacts inherent in the derivation of these metrics.

#### 2.2.3 Computational Tools

All data processing and statistical analyses were conducted in R (R Core Team 2023). Spatial and environmental data were managed using the raster (Hijmans 2021), sf (Pebesma and Bivand 2023) and sp. (Pebesma and Bivand 2005) packages. PCA and hypervolume construction were carried out using the hypervolume (Blonder 2019) and ade4 (Dray and Dufour 2007) packages. SEMs were implemented using piecewiseSEM (Lefcheck 2015), and PCoAs were performed with vegan (Oksanen et al. 2015). Niche originality was estimated using the funrar package (Grenié et al. 2017). Linear models were fitted using the base package while model assumptions were checked using the performance package (Lüdecke et al. 2021) and variable importance assessed using the caret package (Kuhn 2008). Linear mixed‐effect models were fitted using the lme4 package (Bates et al. 2015). Graphical displays were performed using ggplot2 (Wickham 2016).

## 3 Results

### 3.1 Overview of the BioOverlap Database

**FIGURE 2:**
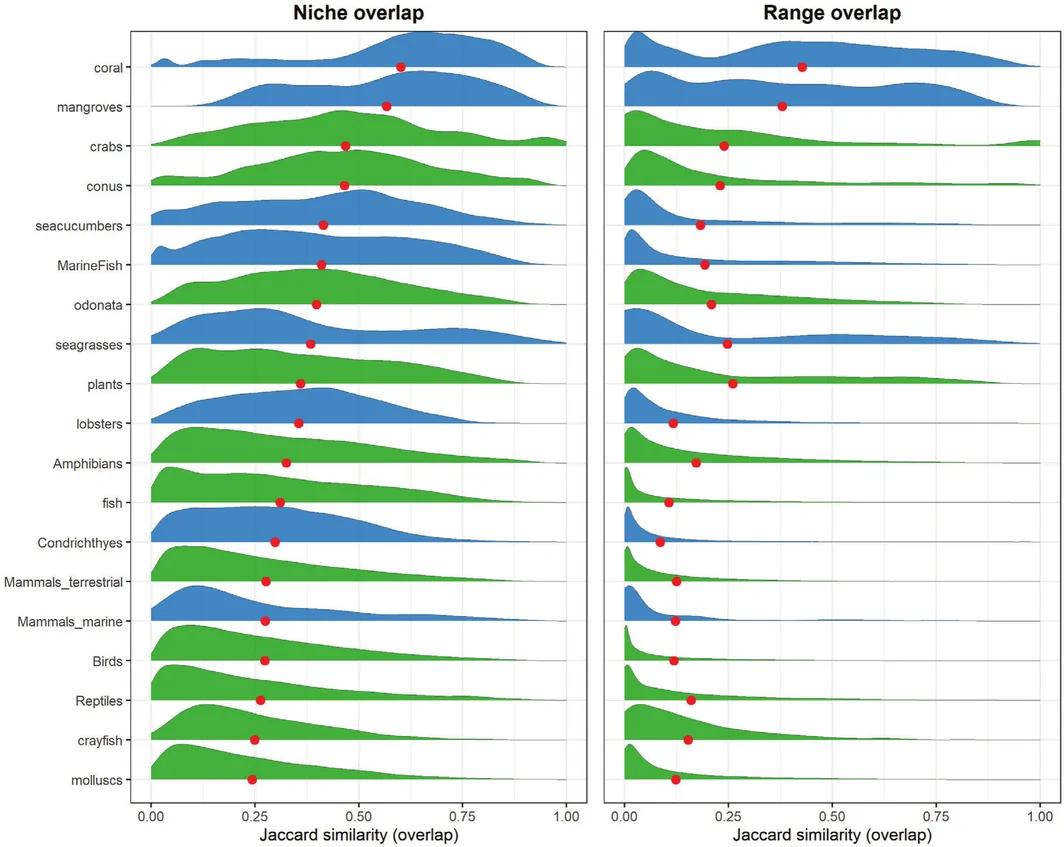
Distributions of niche and geographic overlap among sympatric species pairs across taxonomic groups. Density ridgeline plots show the distribution of (left) climatic niche overlap and (right) geographic range overlap, quantified using Jaccard similarity indices, for each taxonomic group. Taxonomic groups are ordered according to their mean niche overlap values. Red points indicate the mean overlap value for each group. Ridge colors distinguish marine (blue) and terrestrial (green) taxa. Only species pairs with nonzero geographic overlap were included. The distributions illustrate variation in the frequency and magnitude of ecological and geographic similarity among taxonomic groups, with niche overlap providing the primary ranking criterion.

Across all taxonomic groups, niche overlap was higher among species pairs occurring in sympatry (i.e., species pairs with partially overlapping geographic ranges) than among allopatric species pairs (i.e., species pairs with no geographic range overlap; Figure S9). Among sympatric pairs, terrestrial species showed niche overlap values ranging from 0 to 0.96 (mean = 0.27, SD = 0.19), whereas range overlap ranged from values approaching zero to 1 (mean = 0.12, SD = 0.16) (Figure 2; see Table S2 for summary statistics across all species pairs). In marine species, niche overlap ranged from 0 to 0.96 (mean = 0.46, SD = 0.23), while range overlap ranged from values approaching zero to 1 (mean = 0.25, SD = 0.25) (Figure 2). Thus, both niche and range overlap were, on average, higher among marine than terrestrial species pairs, although substantial variation existed among taxonomic groups within each realm (Figure 2). Finally, niche overlap was generally higher between species within the same taxonomic group than between species belonging to different taxonomic groups (Figure S10).

### 3.2 Drivers of Niche Originality

**FIGURE 3:**
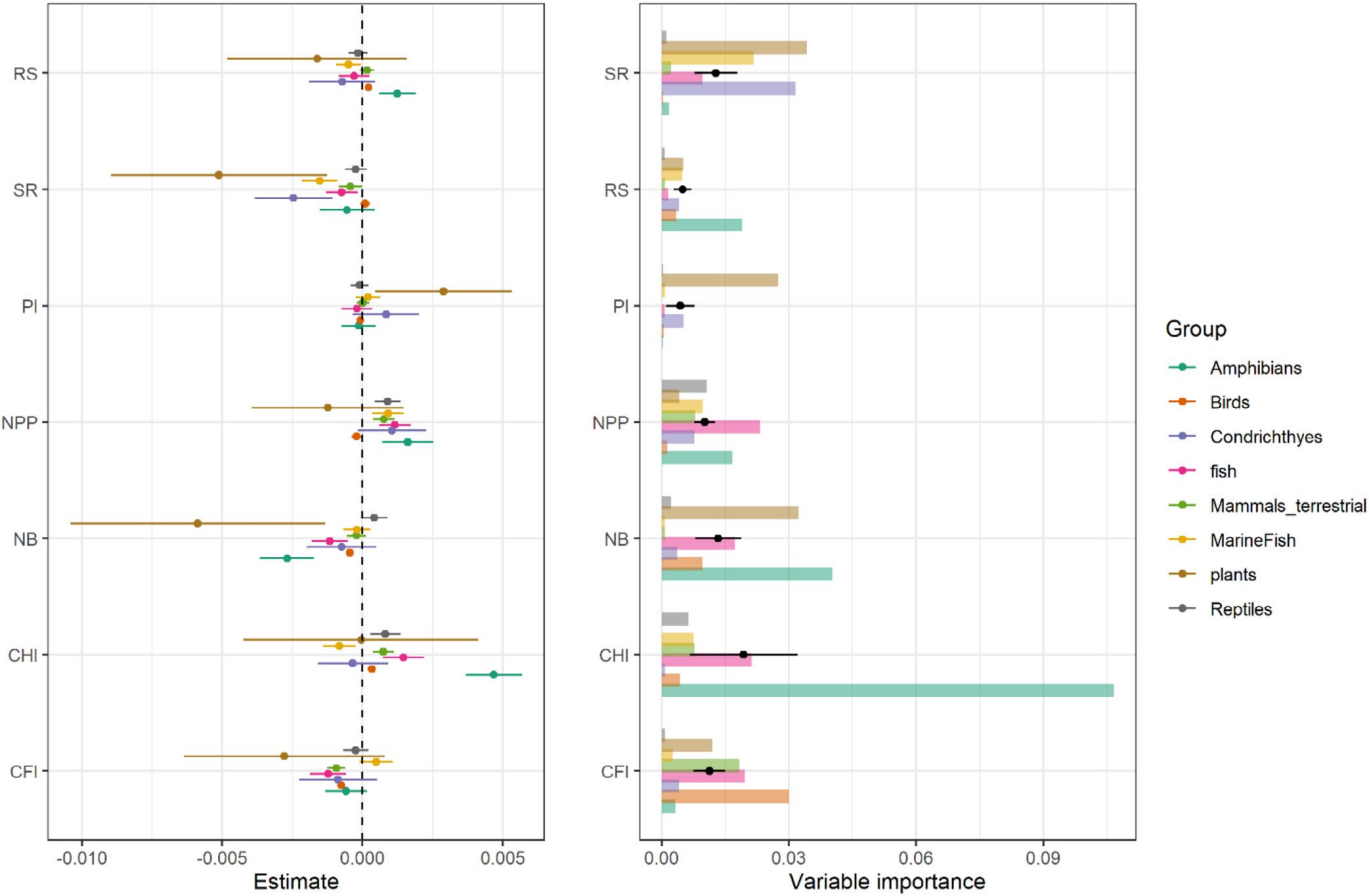
Summary of the effects of environmental and evolutionary predictors on species' niche originality. The left panel shows standardized coefficient estimates from linear models, along with their 95% credible intervals, for each predictor variable. These coefficients represent the direction and strength of each variable's effect on niche originality across species, separately for each taxonomic group. The right panel shows the unique contribution of each predictor to model fit, quantified as partial *R* ^2^ derived from Type II sums of squares. Partial *R* ^2^ represents the proportion of variance explained uniquely by a predictor after accounting for the effects of all other variables in the model. Bars show values for individual taxonomic groups, while black points and horizontal error bars indicate the mean ± standard error across groups. Predictor abbreviations: CFI, climatic frequency intensity (i.e., climatic rarity); CHI, climatic heterogeneity; NB, niche breath; NPP, average net primary productivity within the species range; PI, phylogenetic isolation; SR, average species richness within the species range.

There is no single universal predictor of niche originality across all taxonomic groups, although some common patterns emerge (Figure 3). Phylogenetic isolation generally showed a weak and nonsignificant influence on niche originality, with the exception of plants, for which a positive effect was observed. Range size had limited overall importance, though it showed a positive relationship with niche originality in amphibians and birds, and a negative effect in marine fishes. In contrast, niche breadth emerged as a more consistent and influential driver: species with narrower niches tended to have higher niche originality, reflected in negative effects for amphibians, freshwater fishes, birds, and plants. Species richness also showed a relatively consistent pattern, with negative effects on niche originality detected for freshwater fishes, terrestrial mammals, marine fishes, Chondrichthyes, and plants, suggesting that species in more diverse assemblages may occupy more redundant (or less distinct) niches. Net primary productivity was positively associated with niche originality across most groups, but showed a negative relationship in birds and no significant effect in plants and Chondrichthyes. Climatic rarity tended to reduce niche originality, with negative effects observed in freshwater fishes, birds, and terrestrial mammals. Finally, climatic heterogeneity stood out as one of the most important drivers: it had a strong positive effect on niche originality across most groups except marine fishes, where a negative effect was found, and plants and Chondrichthyes, where the relationship was not statistically significant. Similar results were obtained when niche originality was measured using the ten closest neighbors (Figure S11).

### 3.3 Null‐Model Evaluation of Drivers of Range Overlap

Across taxonomic groups, the models explained a substantial proportion of variation in niche overlap (mean marginal *R* ^2^ = 0.41, SD = 0.08; mean conditional *R* ^2^ = 0.95, SD = 0.01) and geographic range overlap (mean marginal *R* ^2^ = 0.51, SD = 0.12; mean conditional *R* ^2^ = 0.95, SD = 0.11). In contrast, fixed effects explained relatively little variation in maximum niche breadth (mean marginal *R* ^2^ = 0.17, SD = 0.18), despite high overall explanatory power when random effects were included (mean conditional *R* ^2^ = 0.96, SD = 0.01; Table S1).

**FIGURE 4:**
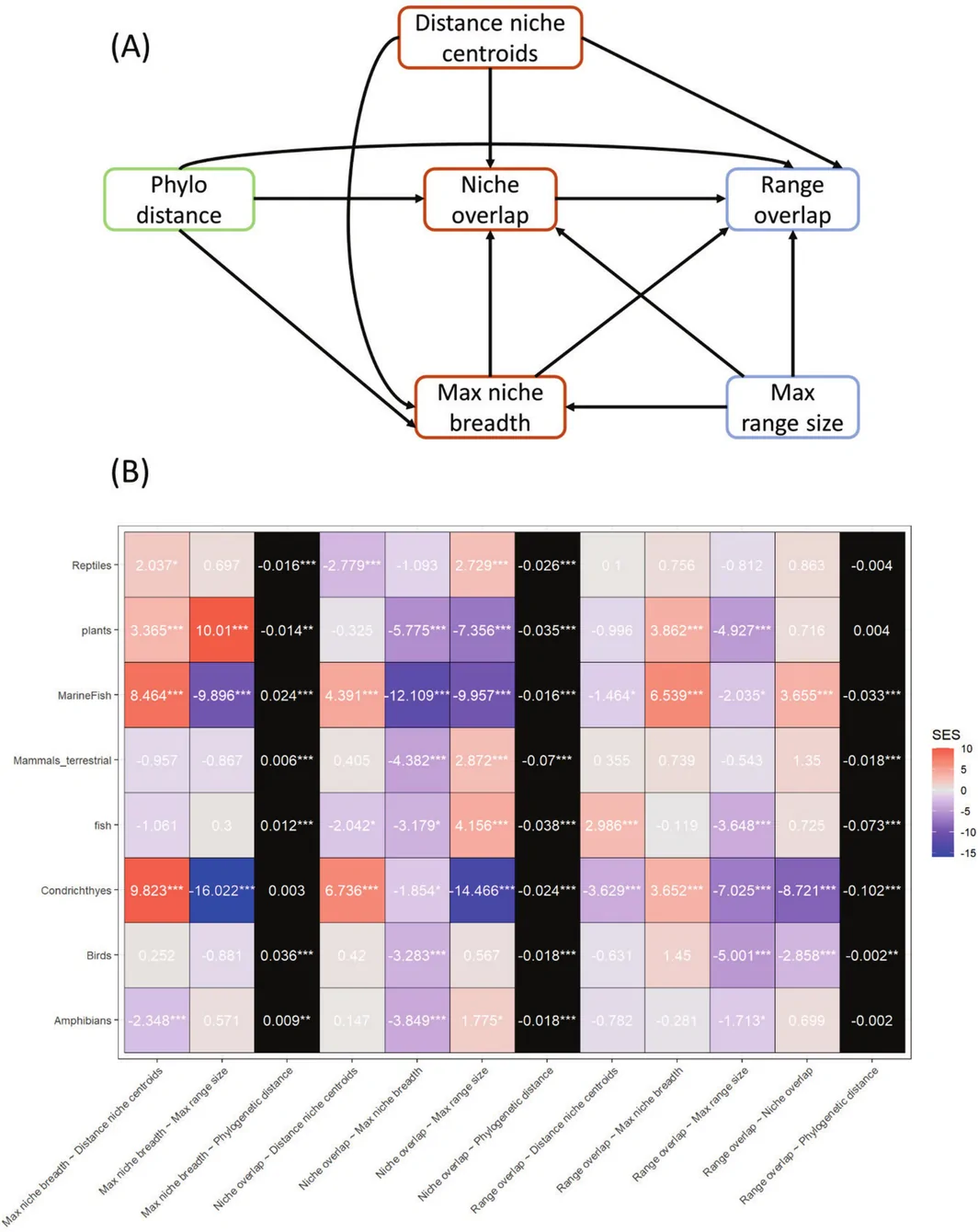
Structural equation model (SEM) framework and results across taxonomic groups. (A) Diagram of the fully saturated structural equation model. Spatial variables are shown in blue, niche‐related variables in orange, and phylogenetic variables in green. Arrows indicate the hypothesized causal relationships tested in the model. (B) Standardized effect sizes (SES) of structural equation model (SEM) paths across multiple taxonomic groups. Tiles show the magnitude and direction of deviation from null expectations for each path, with positive SES in red and negative SES in blue. Paths involving phylogenetic distance are shown in black, displaying the observed effect sizes and associated *p*‐values rather than SES. White text indicates the numerical value of the effect size, with asterisks denoting significance levels (*p < 0.05, **p < 0.01, ***p < 0.001). This figure highlights which relationships deviate from null expectations in amphibians, birds, Chondrichthyes, mammals, plants, marine fishes, and reptiles, illustrating context‐dependent influences on niche and range structure.

Null model comparisons revealed that the relationship between niche overlap and range overlap was, for most taxonomic groups, indistinguishable from random expectations (Figure 4). Standardized effect sizes for the effect of niche overlap on range overlap were generally close to zero and nonsignificant (amphibians: SES = 0.70, mammals: SES = 1.35, reptiles: SES = 0.86, freshwater fish: SES = 0.73, plants: SES = 0.72; all *p* > 0.05), indicating that the observed associations do not depart from those expected under random assembly. This result is consistent with null expectations and with the inherent relationship between environmental and geographic representations of species distributions, indicating that much of the observed association between niche similarity and co‐occurrence may arise independently of ecological structuring. Significant departures from null expectations were nevertheless detected in a subset of taxonomic groups. Birds and Chondrichthyes showed strong negative deviations (birds: SES = −2.86, *p* < 0.001; Chondrichthyes: SES = −8.72, *p* < 0.001), whereas marine fishes exhibited a positive deviation (SES = 3.65, *p* < 0.001). These results indicate that, in these groups, niche–range relationships differ from random expectations, although the direction and magnitude of these deviations vary.

Beyond the focal niche‐range relationship, several additional paths in the structural equation models showed taxon‐specific departures from null expectations, although these patterns were heterogeneous and did not indicate a consistent cross‐taxon signal (Figure 4). For example, niche breadth showed strong nonrandom and negative effects on niche overlap for all groups except reptiles (SES = −1.09), with SES ranging from −1.85 for Condrichthyes to −12.11 for marine fishes. Nonrandom and taxon‐specific effects (both in direction and magnitude) were detected for other predictors of niche and range attributes. Overall, these results point to a context‐dependent structure of associations, where the strength and direction of effects vary among taxonomic groups rather than reflecting a general, cross‐taxon signal.

Because phylogenetic distances were not included in null model simulations, their effects were evaluated from observed SEM standardized coefficients only. Across taxonomic groups, phylogenetic distance showed a consistent negative association with niche overlap, indicating that more closely related species tend to share more similar niches.

## 4 Discussion

Understanding the processes that shape species' environmental originality and patterns of regional co‐occurrence is a central challenge in ecology and biogeography (Chase and Leibold 2003). These patterns not only reflect how species interact with their environment and with each other, but also offer insight into the forces that structure biodiversity across scales (Vellend 2016). Our results can be interpreted within the framework of Hutchinson's duality, which emphasizes the complementary but nonequivalent nature of environmental and geographic representations of biodiversity (Colwell and Rangel 2009). Recent studies have shown that biodiversity patterns may differ substantially depending on whether they are examined in environmental or geographic space (Coelho et al. 2023; Graham et al. 2024). By focusing on pairwise niche overlap and geographic range overlap, our analyses extend this perspective from assemblage‐level biodiversity patterns to species‐level relationships. The strong association observed between environmental similarity and geographic co‐occurrence is consistent with the fact that these representations are intrinsically linked through species distributions, but our null‐model analyses indicate that part of this relationship may arise even in the absence of ecological structuring.

### 4.1 Drivers of Niche Originality

Our results indicate that ecological factors play a primary role in shaping niche originality, whereas evolutionary uniqueness has a limited influence. Across most taxonomic groups, phylogenetic isolation had weak or nonsignificant effects, with the exception of plants, where evolutionarily distinct species tended to occupy more unique climatic niches. This suggests that phylogenetic isolation alone does not reliably predict ecological distinctiveness across broad climatic dimensions, challenging the generality of the “rarity begets rarity” hypothesis (Ong et al. 2022) and highlighting the partial decoupling of evolutionary and ecological distinctiveness.

Among ecological drivers, niche breadth consistently emerged as a strong predictor: species with narrow niches tended to show higher originality across multiple taxa, supporting the idea that specialization promotes occupancy of unique environmental conditions (Slatyer et al. 2013). Interestingly, no strong counter‐examples (i.e., positive effects of niche breadth) were found, suggesting that generalism rarely fosters ecological distinctiveness at macroclimatic scales. Climatic heterogeneity also played an important role, with generally positive effects in terrestrial systems, suggesting that environmentally complex regions promote niche originality (Stein et al. 2014; Enquist et al. 2019). In contrast, this pattern was weak or reversed in several marine groups, highlighting fundamental differences in how environmental variability structures niches in aquatic versus terrestrial systems (Chase and Leibold 2003). Unlike terrestrial systems, where heterogeneity can create distinct microhabitats, the ocean's fluid and interconnected nature may lead to environmental homogenization at broader scales (Steele 1991). In such settings, species may evolve broader, overlapping niches to cope with fluctuating conditions, reducing niche originality. Additionally, strong dispersal and fewer physical barriers in marine systems could limit the spatial isolation needed for ecological specialization to emerge (Kinlan and Gaines 2003).

Net primary productivity generally had a positive effect on niche originality, consistent with resource‐rich environments fostering diversification and specialization (Chesson 2000; Evans et al. 2005). However, this relationship was negative in birds, likely reflecting their high mobility and decoupling from local resource constraints. This pattern is consistent with the hypothesis that avian niches broadly overlap around a core set of abundant resources, for which a generalized morphology is favored (the “central tendency” hypothesis), while specialization occurs primarily at the niche periphery through the exploitation of more exclusive resources (Ricklefs 2012). Nonsignificant relationships were observed for plants and Chondrichthyes. These group‐specific responses highlight that mobility, life history, and trophic strategy can modulate the productivity‐originality relationship. Similarly, range size had inconsistent effects, reflecting differences in how species experience habitat complexity and environmental variability across systems (Slatyer et al. 2013).

In contrast, species richness consistently showed negative effects on niche originality across many groups, suggesting that niche space may be more saturated in species‐rich assemblages, limiting opportunities for ecological distinctiveness (MacArthur and Levins 1967b). Yet, ecological differentiation may occur at finer spatial scales or through biotic and environmental factors not captured by broad macroclimatic variables (e.g., microhabitats, resource use, or biotic interactions) allowing coexistence without strong climatic niche divergence (Chesson 2000). Finally, climatic rarity had negative effects on niche originality in several terrestrial groups suggesting that species occupying climatically rare environments tended to exhibit greater niche originality than species associated with common climatic conditions.

Overall, these results show that ecological variables, particularly niche breadth, climatic heterogeneity, and net primary productivity, play a more central role in shaping niche originality than evolutionary uniqueness. While phylogenetic isolation matters in some groups (notably plants), it is not a general driver. Importantly, we found consistent negative effects of species richness, highlighting potential limits to ecological distinctiveness in crowded niche spaces. The taxon‐specific responses, especially between marine and terrestrial groups, also underscore the importance of ecological context and habitat type in determining how originality arises and persists, echoing findings in other fields (Pinsky et al. 2019).

### 4.2 Drivers of Regional Coexistence

Analyses based on null model simulations revealed that much of the apparent association between niche similarity and geographic co‐occurrence can arise from statistical structure. Positive correlations between niche overlap and range overlap frequently emerged under random expectations, reflecting the shared dependence of both metrics on species' geographic distributions and the structure of environmental space. This result highlights the importance of evaluating observed patterns against appropriate null models to avoid overinterpreting spurious signals (Peterson and Soberón 2012; Warren et al. 2014).

Despite this dominant null signal, a subset of clades exhibited meaningful departures from random expectations. Birds and Chondrichthyes showed negative SES, indicating that ecologically similar species co‐occur less than expected based on niche similarity. This pattern is consistent with the action of competitive exclusion, historical constraints, or dispersal limitations preventing coexistence among niche‐similar species (Macarthur and Levins 1967a; Chase and Leibold 2003). Conversely, marine fishes showed a positive SES, with ecologically similar species co‐occurring more than expected, suggesting that environmental filtering or shared habitat preferences can promote co‐occurrence in certain contexts (Webb et al. 2002). These deviations illustrate that, while the general niche‐range relationship is largely shaped by statistical structure, ecological processes can still generate detectable signals in specific taxa.

Other predictors included in the SEM showed variable departures from null expectations. While several relationships remained weak or indistinguishable from random in some groups (e.g., amphibians and mammals), others displayed strong and consistent effects among taxon. In particular, the negative association between niche breadth and niche overlap suggests that some macroclimatic generalists may occupy distinct regions of environmental space rather than exhibiting uniformly high overlap with other species. In contrast, climatic specialists tend to overlap strongly in macroclimatic niche space and may therefore rely on differentiation along finer‐scale environmental axes or biotic niche dimensions (such as microhabitat use, resource specialization or phenology) to limit competition and facilitate coexistence. These results support a scale‐dependent view of niche specialization (Devictor et al. 2010), whereby generalist species differentiate primarily at broad macroclimatic scales, while specialists, despite strong overlap in coarse climatic space, may segregate along finer environmental or biotic niche dimensions not captured by climatic niches (Slatyer et al. 2013). Similarly, phylogenetic distance showed a consistent negative effect on niche overlap among all groups, indicating that more closely related species tend to occupy more similar niches, in line with patterns of phylogenetic niche conservatism (Losos 2008; Wiens et al. 2010). Multiple paths involving niche overlap and range overlap also showed strong deviations in marine fishes, Chondrichthyes, and plants, highlighting the importance of ecological context in shaping these relationships (Wiens 2011). Indirect effects mediated through niche centroid distance were generally consistent with null expectations, further emphasizing that not all components of the modeled network contribute equally to deviations from randomness.

Importantly, these findings do not imply that niche similarity is irrelevant for coexistence when inferred from macroclimatic niches estimated from distribution data, but rather that its interpretation requires explicit comparison to null expectations. Descriptive patterns, such as higher niche overlap among sympatric species or within taxonomic groups, primarily reflect the underlying association between environmental similarity and spatial co‐occurrence and therefore do not, on their own, provide evidence for ecological processes. Instead, it is the deviation from null expectations that identifies the contexts in which mechanisms such as environmental filtering, competition, or historical contingency may operate. Integrating independent estimates of species' niches (i.e., estimates derived from information other than the geographic distribution data used to quantify niche overlap, such as physiological tolerances, functional traits, or experimental data) will be critical to validate these inferences and disentangle genuine ecological mechanisms from methodological artifacts (Kearney and Porter 2009; Blonder et al. 2014).

Overall, our results demonstrate that large‐scale patterns of regional coexistence are shaped both by the statistical structure of species distributions and by ecological or evolutionary processes in specific clades. Null model frameworks, applied alongside independent niche data, provide a robust approach for distinguishing signal from artifact and refining our understanding of the drivers of species co‐occurrence.

### 4.3 Limitations and Future Directions

While BioOverlap provides a powerful framework for investigating macroecological patterns, several limitations of the database should be acknowledged. First, the use of IUCN range maps, while standardized and globally available, may overestimate range overlap and miss fine‐scale ecological partitioning, particularly in diverse, fragmented, or environmentally heterogeneous landscapes (Hurlbert and Jetz 2007). Second, the database focuses exclusively on climatic dimensions of the niche, omitting biotic interactions, trophic roles, and life‐history strategies that often play critical roles in species coexistence and exclusion (Violle et al. 2017; Hood et al. 2021). An additional limitation concerns statistical dependence among pairwise observations. Because species participate in multiple dyads, observations are not fully independent. We accounted for repeated participation using species identity as a random effect and explicitly included phylogenetic distance as a predictor. However, this approach does not fully model the covariance structure expected from shared evolutionary history among species pairs. Existing phylogenetic comparative methods, such as phylogenetic generalized least squares (PGLS; Freckleton et al. 2002), are designed for species‐level response variables and rely on a covariance matrix linking individual taxa, making them difficult to apply directly to dyadic observations. Similarly, species distributions are spatially and biogeographically structured, which may generate residual dependence among species pairs beyond that captured by range overlap and geographic distance (Legendre 1993; Dormann et al. 2007). Consequently, some residual phylogenetic or spatial nonindependence may remain in our analyses. Future developments based on network and dyadic modeling approaches, multi‐membership mixed models, or explicit phylogenetic models for pairwise data could provide a more rigorous treatment of these dependencies (Ives and Godfray 2006; Hoff 2011).

Despite these caveats, BioOverlap represents a scalable and versatile platform for exploring how climate, evolutionary history, and environmental variation shape biodiversity patterns at large spatial scales. Integrating BioOverlap with species distribution models, extinction scenarios, or paleoecological data can help track how niche and range overlap patterns have changed over time or may shift under future climate change (see Box 1 for potential applications). Such approaches enable assessments of functional redundancy loss, the emergence of novel species interactions, and community reassembly under global change scenarios (Manlick and Pauli 2020; Carmona et al. 2021). Future extensions could incorporate independent estimates of niches based on physiological tolerances, functional traits, or experimental manipulations (Kearney and Porter 2009; Blonder et al. 2014), allowing for a more mechanistic understanding of the processes underlying regional coexistence and environmental specialization.

## 5 Conclusion

By combining global‐scale data on species distributions and climate, the BioOverlap framework provides a unified approach to quantify niche originality, niche overlap, and geographic co‐occurrence across taxa. Our analyses reveal a clear contrast in the inferential power of these metrics: while species‐level properties such as niche originality capture consistent ecological signals, pairwise relationships between niche overlap and range overlap are, in most cases, indistinguishable from null expectations. Viewed through the lens of Hutchinson's duality, our results suggest that translating patterns between environmental and geographic space requires particular caution when both representations are derived from the same underlying data. Environmental similarity and geographic co‐occurrence are intrinsically connected, yet the strength of this connection varies among taxa and often does not exceed null expectations. This highlights the importance of explicitly evaluating whether observed relationships reflect ecological structure or simply emerge from the geometry of species distributions and environmental availability. As such, this study provides a general framework for distinguishing when large‐scale niche patterns reflect robust ecological signals and when they are more likely to emerge from stochastic or methodological effects. Beyond the specific results presented here, BioOverlap opens new opportunities for integrative analyses of niche structure and species overlap across systems, while clarifying the conditions under which niche‐based inferences can be considered reliable (Box 1).

## Author Contributions

**Mathieu Chevalier:** conceptualization (lead), data curation (lead), formal analysis (lead), investigation (lead), methodology (lead), project administration (lead), validation (lead), visualization (lead), writing – original draft (lead), writing – review and editing (equal). **Clement Violet:** data curation (supporting), formal analysis (supporting), resources (equal), visualization (supporting), writing – review and editing (supporting). **Aurélien Boyé:** conceptualization (equal), formal analysis (equal), methodology (equal), visualization (equal), writing – original draft (equal), writing – review and editing (equal).

## Funding

This work was supported by the CBD project, funded through ISblue, the Interdisciplinary Graduate School for the Blue Planet (ANR‐17‐EURE‐0015), and co‐funded by the French government under the “Investissements d'Avenir” programme, as part of France 2030. C.V. was supported by a PhD fellowship funded by Ifremer.

## Conflicts of Interest

The authors declare no conflicts of interest.

## Supporting information


**Text S1:** SEM diagnostics.
**Table S1:** Summary of model fit and diagnostic statistics for component models of the piecewise structural equation models across taxonomic groups. Marginal and conditional *R* ^2^ values quantify variance explained by fixed effects and by fixed plus random effects, respectively. Residual skewness and kurtosis, Breusch–Pagan (BP) test statistics, BP *p*‐values, and maximum variance inflation factors (VIF) are provided for diagnostic assessment. Given the exceptionally large sample sizes of several datasets, Breusch–Pagan tests had high statistical power and detected statistically significant departures from homoscedasticity even when deviations were modest. Therefore, evidence for heteroscedasticity was interpreted based on the magnitude of residual patterns and visual inspection of diagnostic plots rather than statistical significance alone.
**Table S2:** Summary statistics for different measures present in the BioOverlap database obtained from species‐wise estimates of 3D niche hypervolumes and 2D range polygons.
**Figure S1:** Residual diagnostic plots for the piecewise structural equation models fitted on Amphibians. Each row corresponds to one component model of the SEM, representing (from top to bottom) range overlap, niche overlap, and maximum niche breadth (log transform). Columns show (from left to right): residuals versus fitted values, normal Q–Q plots, scale‐location plots, and residuals versus the main predictor. Red lines represent locally estimated smoothing trends, and dashed horizontal lines indicate zero residuals. For computational efficiency, datasets containing more than 10,000 observations were randomly subsampled for visualization only; model fitting was performed using the complete datasets. These diagnostics were used to assess residual structure, normality, variance homogeneity, and potential model misspecification across SEM component models.
**Figure S2:** Residual diagnostic plots for the piecewise structural equation models fitted on Birds. Each row corresponds to one component model of the SEM, representing (from top to bottom) range overlap, niche overlap, and maximum niche breadth (log transform). Columns show (from left to right): residuals versus fitted values, normal Q–Q plots, scale‐location plots, and residuals versus the main predictor. Red lines represent locally estimated smoothing trends, and dashed horizontal lines indicate zero residuals. For computational efficiency, datasets containing more than 10,000 observations were randomly subsampled for visualization only; model fitting was performed using the complete datasets. These diagnostics were used to assess residual structure, normality, variance homogeneity, and potential model misspecification across SEM component models.
**Figure S3:** Residual diagnostic plots for the piecewise structural equation models fitted on Condrichthyes. Each row corresponds to one component model of the SEM, representing (from top to bottom) range overlap, niche overlap, and maximum niche breadth (log transform). Columns show (from left to right): residuals versus fitted values, normal Q–Q plots, scale‐location plots, and residuals versus the main predictor. Red lines represent locally estimated smoothing trends, and dashed horizontal lines indicate zero residuals. For computational efficiency, datasets containing more than 10,000 observations were randomly subsampled for visualization only; model fitting was performed using the complete datasets. These diagnostics were used to assess residual structure, normality, variance homogeneity, and potential model misspecification across SEM component models.
**Figure S4:** Residual diagnostic plots for the piecewise structural equation models fitted on Freshwater Fish. Each row corresponds to one component model of the SEM, representing (from top to bottom) range overlap, niche overlap, and maximum niche breadth (log transform). Columns show (from left to right): residuals versus fitted values, normal Q–Q plots, scale‐location plots, and residuals versus the main predictor. Red lines represent locally estimated smoothing trends, and dashed horizontal lines indicate zero residuals. For computational efficiency, datasets containing more than 10,000 observations were randomly subsampled for visualization only; model fitting was performed using the complete datasets. These diagnostics were used to assess residual structure, normality, variance homogeneity, and potential model misspecification across SEM component models.
**Figure S5:** Residual diagnostic plots for the piecewise structural equation models fitted on Terrestrial Mammals. Each row corresponds to one component model of the SEM, representing (from top to bottom) range overlap, niche overlap, and maximum niche breadth (log transform). Columns show (from left to right): residuals versus fitted values, normal Q–Q plots, scale‐location plots, and residuals versus the main predictor. Red lines represent locally estimated smoothing trends, and dashed horizontal lines indicate zero residuals. For computational efficiency, datasets containing more than 10,000 observations were randomly subsampled for visualization only; model fitting was performed using the complete datasets. These diagnostics were used to assess residual structure, normality, variance homogeneity, and potential model misspecification across SEM component models.
**Figure S6:** Residual diagnostic plots for the piecewise structural equation models fitted on Marine Fish. Each row corresponds to one component model of the SEM, representing (from top to bottom) range overlap, niche overlap, and maximum niche breadth (log transform). Columns show (from left to right): residuals versus fitted values, normal Q–Q plots, scale‐location plots, and residuals versus the main predictor. Red lines represent locally estimated smoothing trends, and dashed horizontal lines indicate zero residuals. For computational efficiency, datasets containing more than 10,000 observations were randomly subsampled for visualization only; model fitting was performed using the complete datasets. These diagnostics were used to assess residual structure, normality, variance homogeneity, and potential model misspecification across SEM component models.
**Figure S7:** Residual diagnostic plots for the piecewise structural equation models fitted on Plants. Each row corresponds to one component model of the SEM, representing (from top to bottom) range overlap, niche overlap, and maximum niche breadth (log transform). Columns show (from left to right): residuals versus fitted values, normal Q–Q plots, scale‐location plots, and residuals versus the main predictor. Red lines represent locally estimated smoothing trends, and dashed horizontal lines indicate zero residuals. For computational efficiency, datasets containing more than 10,000 observations were randomly subsampled for visualization only; model fitting was performed using the complete datasets. These diagnostics were used to assess residual structure, normality, variance homogeneity, and potential model misspecification across SEM component models.
**Figure S8:** Residual diagnostic plots for the piecewise structural equation models fitted on Reptiles. Each row corresponds to one component model of the SEM, representing (from top to bottom) range overlap, niche overlap, and maximum niche breadth (log transform). Columns show (from left to right): residuals versus fitted values, normal Q–Q plots, scale‐location plots, and residuals versus the main predictor. Red lines represent locally estimated smoothing trends, and dashed horizontal lines indicate zero residuals. For computational efficiency, datasets containing more than 10,000 observations were randomly subsampled for visualization only; model fitting was performed using the complete datasets. These diagnostics were used to assess residual structure, normality, variance homogeneity, and potential model misspecification across SEM component models.
**Figure S9:** Boxplots highlighting niche overlap between species that are found in sympatry or in allopatry for the different taxonomic groups present in the BioOverlap database.
**Figure S10:** Boxplots highlighting niche overlap between species within the same taxonomic group (Within) or in different taxonomic groups (Between).
**Figure S11:** Sensitivity analysis related to the drivers of niche originality when measured using the mean distance to the ten closest neighbors in the environmental space. For further details, see Figure 3 in the main text.

## Data Availability

All the data used in this study are publicly available: IUCN range maps can be found at https://www.iucnredlist.org/resources/spatial‐data‐download; Terrestrial climatic data can be found at https://chelsa‐climate.org/; Martine climatic data can be found at https://www.bio‐oracle.org/. The BioOverlap database is freely available at the following address https://doi.org/10.17882/101615. The R code and data (IUCN range maps, phylogenetic data, environmental PCA and range features) used to build the database and conduct analyses related to niche originality and regional coexistence can be found at https://zenodo.org/records/5113900.

## References

[ece374215-bib-0001] Arita, T. , J. Andre , P. Rodrı , and J. Sobero . 2008. “Species Diversity and Distribution in Presence‐Absence Matrices: Mathematical Relationships and.” American Naturalist 172: 519–532. 10.1086/590954 18729736

[ece374215-bib-0002] Assis, J. , L. Tyberghein , S. Bosch , H. Verbruggen , E. A. Serrão , and O. De Clerck . 2018. “Bio‐ORACLE v2.0: Extending Marine Data Layers for Bioclimatic Modelling.” Global Ecology and Biogeography 27: 277–284.

[ece374215-bib-0003] Austin, M. P. 2002. “Spatial Prediction of Species Distribution: An Interface Between Ecological Theory and Statistical Modelling.” Ecological Modelling 6157: 101–118.

[ece374215-bib-0004] Barabás, G. , R. D'Andrea , and S. M. Stump . 2018. “Chesson's Coexistence Theory.” Ecological Monographs 88: 277–303.

[ece374215-bib-0005] Bates, D. , M. Mächler , B. Bolker , and S. Walker . 2015. “Fitting Linear Mixed‐Effects Models Using {lme4}.” Journal of Statistical Software 67: 1–48.

[ece374215-bib-0006] Bates, O. K. , and C. Bertelsmeier . 2021. “Climatic Niche Shifts in Introduced Species.” Current Biology 31: R1252–R1266. 34637738 10.1016/j.cub.2021.08.035

[ece374215-bib-0007] Blonder, B. 2019. “Hypervolume: High Dimensional Geometry and Set Operations Using Kernel Density Estimation, Support Vector Machines, and Convex Hulls”.

[ece374215-bib-0008] Blonder, B. , C. Lamanna , C. Violle , and B. J. Enquist . 2014. “The n‐Dimensional Hypervolume.” Global Ecology and Biogeography 23: 595–609.

[ece374215-bib-0009] Broennimann, O. , M. C. Fitzpatrick , P. B. Pearman , et al. 2012. “Measuring Ecological Niche Overlap From Occurrence and Spatial Environmental Data.” Global Ecology and Biogeography 21: 481–497.

[ece374215-bib-0010] Carmona, C. P. , R. Tamme , M. Pärtel , et al. 2021. “Erosion of Global Functional Diversity Across the Tree of Life.” Science Advances 7: eabf2675. 33771870 10.1126/sciadv.abf2675 PMC7997514

[ece374215-bib-0011] Carroll, G. , K. K. Holsman , S. Brodie , et al. 2019. “A Review of Methods for Quantifying Spatial Predator–Prey Overlap.” Global Ecology and Biogeography 28: 1561–1577.

[ece374215-bib-0012] Carvalho, J. C. , and P. Cardoso . 2020. “Decomposing the Causes for Niche Differentiation Between Species Using Hypervolumes.” Frontiers in Ecology and Evolution 8: 1–7.

[ece374215-bib-0013] Chase, J. M. , and M. A. Leibold . 2003. Ecological Niches: Linking Classical and Contemporary Approaches. University of Chicago Press.

[ece374215-bib-0014] Chesson, P. 2000. “Mechanisms of Maintenance of Species Diversity.” Annual Review of Ecology and Systematics 31: 343–366.

[ece374215-bib-0015] Chevalier, M. , A. Boyé , and C. Violet . 2024. BioOverlap – A Database of Niche and Range Overlap Measures Between Species at the Global Scale. SEANOE. 10.17882/101615.

[ece374215-bib-0016] Coelho, M. T. P. , E. Barreto , T. F. Rangel , et al. 2023. “The Geography of Climate and the Global Patterns of Species Diversity.” Nature 622: 537–544. 37758942 10.1038/s41586-023-06577-5 PMC10584679

[ece374215-bib-0017] Colwell, R. K. , and D. Futuyama . 1971. “On the Measurement of Niche Breadth and Overlap.” Ecology 52: 567–576. 28973805 10.2307/1934144

[ece374215-bib-0018] Colwell, R. K. , and D. C. Lees . 2000. “The Mid‐Domain Effect: Geometric Constraints on the Geography of Species Richness.” Trends in Ecology & Evolution 15: 70–76. 10652559 10.1016/s0169-5347(99)01767-x

[ece374215-bib-0019] Colwell, R. K. , and T. F. Rangel . 2009. “Hutchinson's Duality: The Once and Future Niche.” Proceedings of the National Academy of Sciences of the United States of America 106: 19651–19658. 19805163 10.1073/pnas.0901650106 PMC2780946

[ece374215-bib-0020] Davies, J. T. , S. Meiri , T. G. Barraclough , and J. L. Gittleman . 2007. “Species Co‐Existence and Character Divergence Across Carnivores.” Ecology Letters 10: 146–152. 17257102 10.1111/j.1461-0248.2006.01005.x

[ece374215-bib-0021] Devictor, V. , D. Mouillot , C. Meynard , F. Jiguet , W. Thuiller , and N. Mouquet . 2010. “Spatial Mismatch and Congruence Between Taxonomic, Phylogenetic and Functional Diversity: The Need for Integrative Conservation Strategies in a Changing World.” Ecology Letters 13: 1030–1040. 20545736 10.1111/j.1461-0248.2010.01493.x

[ece374215-bib-0022] Dormann, C. , J. McPherson , M. Araújo , et al. 2007. “Methods to Account for Spatial Autocorrelation in the Analysis of Species Distributional Data: A Review.” Ecography 30: 609–628.

[ece374215-bib-0023] Dray, S. , and A. B. Dufour . 2007. “The ade4 Package: Implementing the Duality Diagram for Ecologists.” Journal of Statistical Software 22: 1–20.

[ece374215-bib-0024] Enquist, B. J. , X. Feng , B. Boyle , et al. 2019. “The Commonness of Rarity: Global and Future Distribution of Rarity Across Land Plants.” Science Advances 5: 1–13. 10.1126/sciadv.aaz0414 PMC6881168 31807712

[ece374215-bib-0025] Evans, K. L. , P. H. Warren , and K. J. Gaston . 2005. “Species‐Energy Relationships at the Macroecological Scale: A Review of the Mechanisms.” Biological Reviews of the Cambridge Philosophical Society 80: 1–25. 15727036 10.1017/s1464793104006517

[ece374215-bib-0026] Fournier, B. , H. Vázquez‐Rivera , S. Clappe , L. Donelle , P. H. P. Braga , and P. R. Peres‐Neto . 2020. “The Spatial Frequency of Climatic Conditions Affects Niche Composition and Functional Diversity of Species Assemblages: The Case of Angiosperms.” Ecology Letters 23: 254–264. 31749270 10.1111/ele.13425

[ece374215-bib-0027] Freckleton, R. , P. Harvey , and M. Pagel . 2002. “Phylogenetic Analysis and Comparative Data: A Test and Review of Evidence.” American Naturalist 160: 712–726. 10.1086/343873 18707460

[ece374215-bib-0028] Garrido, M. , S. K. Hansen , R. Yaari , and H. Hawlena . 2022. “A Model Selection Approach to Structural Equation Modelling: A Critical Evaluation and a Road Map for Ecologists.” Methods in Ecology and Evolution 13: 42–53.

[ece374215-bib-0029] Geange, S. W. , S. Pledger , K. C. Burns , and J. S. Shima . 2011. “A Unified Analysis of Niche Overlap Incorporating Data of Different Types.” Methods in Ecology and Evolution 2: 175–184.

[ece374215-bib-0030] Godsoe, W. , J. Jankowski , R. D. Holt , and D. Gravel . 2017. “Integrating Biogeography With Contemporary Niche Theory.” Trends in Ecology & Evolution 32: 488–499. 28477957 10.1016/j.tree.2017.03.008

[ece374215-bib-0031] Godwin, C. M. , F. H. Chang , and B. J. Cardinale . 2020. “An Empiricist's Guide to Modern Coexistence Theory for Competitive Communities.” Oikos 129: 1109–1127.

[ece374215-bib-0032] Gotelli, N. J. , and G. R. Graves . 1996. Null Models in Ecology. Smithsonian Institution Press.

[ece374215-bib-0033] Gouveia, S. F. , J. Hortal , M. Tejedo , et al. 2014. “Climatic Niche at Physiological and Macroecological Scales: The Thermal Tolerance‐Geographical Range Interface and Niche Dimensionality.” Global Ecology and Biogeography 23: 446–456.

[ece374215-bib-0034] Grace, J. B. 2006. Structural Equation Modeling and Natural Systems. Cambridge University Press.

[ece374215-bib-0035] Graham, C. H. , M. Lima , A. Elisa , et al. 2024. “Biodiversity Patterns Redefined in Environmental Space.” Ecology Letters 28: e70008. 10.1111/ele.70008 39968787

[ece374215-bib-0036] Grenié, M. , P. Denelle , C. M. Tucker , F. Munoz , and C. Violle . 2017. “Funrar: An R Package to Characterize Functional Rarity.” Diversity and Distributions 23: 1365–1371.

[ece374215-bib-0037] Guisan, A. , W. Thuiller , and N. E. Zimmermann . 2017. Habitat Suitability and Distribution Models: With Applications in R. *Habitat Suitabil. Distrib. Model. With Appl. R*. 1st ed. Cambridge University Press.

[ece374215-bib-0038] Hijmans, R. J. 2021. “raster: Geographic Data Analysis and Modeling”.

[ece374215-bib-0039] HilleRisLambers, J. , P. B. Adler , W. S. Harpole , J. M. Levine , and M. M. Mayfield . 2012. “Rethinking Community Assembly Through the Lens of Coexistence Theory.” Annual Review of Ecology, Evolution, and Systematics 43: 227–248.

[ece374215-bib-0040] Hoff, P. D. 2011. “Bilinear Mixed‐Effects Models for Dyadic Data Bilinear Mixed‐Effects Models for Dyadic Data.” Journal of the American Statistical Association 1459: 286–295.

[ece374215-bib-0041] Hood, G. R. , D. Blankinship , M. M. Doellman , and J. L. Feder . 2021. “Temporal Resource Partitioning Mitigates Interspecific Competition and Promotes Coexistence Among Insect Parasites.” Biological Reviews 96: 1969–1988. 34041840 10.1111/brv.12735

[ece374215-bib-0042] Hortal, J. , F. De Bello , J. A. F. Diniz‐Filho , T. M. Lewinsohn , J. M. Lobo , and R. J. Ladle . 2015. “Seven Shortfalls That Beset Large‐Scale Knowledge of Biodiversity.” Annual Review of Ecology, Evolution, and Systematics 46: 523–549.

[ece374215-bib-0043] Hurlbert, A. H. , and W. Jetz . 2007. “Species Richness, Hotspots, and the Scale Dependence of Range Maps in Ecology and Conservation.” Proceedings of the National Academy of Sciences of the United States of America 104: 13384–13389. 17686977 10.1073/pnas.0704469104 PMC1948922

[ece374215-bib-0044] Hutchinson, G. 1957. “Concluding Remarks.” Cold Spring Harbor Symposia on Quantitative Biology 22: 415–427.

[ece374215-bib-0045] Ives, A. R. , and H. C. J. Godfray . 2006. “Phylogenetic Analysis of Trophic Associations.” American Naturalist 168: E1–E14. 10.1086/505157 16874610

[ece374215-bib-0046] Jackson, S. T. , and J. T. Overpeck . 2000. “Responses of Plant Populations and Communities to Environmental Changes of the Late Quaternary.” Paleobiology 26: 194–220.

[ece374215-bib-0047] Jetz, W. , and R. A. Pyron . 2018. “The Interplay of Past Diversification and Evolutionary Isolation With Present Imperilment Across the Amphibian Tree of Life.” Nature Ecology & Evolution 2: 850–858. 29581588 10.1038/s41559-018-0515-5

[ece374215-bib-0048] Jetz, W. , G. H. Thomas , J. B. Joy , K. Hartmann , and A. O. Mooers . 2012. “The Global Diversity of Birds in Space and Time.” Nature 491: 444–448. 23123857 10.1038/nature11631

[ece374215-bib-0049] Karger, D. N. , O. Conrad , J. Böhner , et al. 2017. “Climatologies at High Resolution for the Earth's Land Surface Areas.” Scientific Data 4: 1–20. 10.1038/sdata.2017.122 PMC5584396 28872642

[ece374215-bib-0050] Kearney, M. , and W. Porter . 2009. “Mechanistic Niche Modelling: Combining Physiological and Spatial Data to Predict Species' Ranges.” Ecology Letters 12: 334–350. 19292794 10.1111/j.1461-0248.2008.01277.x

[ece374215-bib-0051] Kerner, E. H. 1974. “Why Are There So Many Species?” Bulletin of Mathematical Biology 36: 477–488. 4457195 10.1007/BF02463261

[ece374215-bib-0052] Kinlan, B. P. , and S. D. Gaines . 2003. “Propagule Dispersal in Marine and Terrestrial Environments: A Community Perspective.” Ecology 84: 2007–2020.

[ece374215-bib-0053] Kondratyeva, A. , P. Grandcolas , and S. Pavoine . 2019. “Reconciling the Concepts and Measures of Diversity, Rarity and Originality in Ecology and Evolution.” Biological Reviews 94: 1317–1337. 30861626 10.1111/brv.12504 PMC6850657

[ece374215-bib-0054] Kuhn, M. 2008. “Building Predictive Models in R Using the Caret Package.” Journal of Statistical Software 28: 1–26.27774042

[ece374215-bib-0055] Lefcheck, J. S. 2015. “piecewiseSEM: Piecewise Structural Equation Modelling in r for Ecology, Evolution, and Systematics.” Methods in Ecology and Evolution 7: 1–20.

[ece374215-bib-0056] Legendre, P. 1993. “Spatial Autocorrelation: Trouble or New Paradigm?” Ecology 74: 1659–1673.

[ece374215-bib-0057] Legendre, P. , and L. F. J. Legendre . 2012. Numerical Ecology. 3rd ed. Elsevier.

[ece374215-bib-0058] Leibold, M. A. 1995. “The Niche Concept Revisited: Mechanistic Models and Community Context.” Ecology 76: 1371–1382.

[ece374215-bib-0059] Lesser, J. S. , W. R. James , C. D. Stallings , R. M. Wilson , and J. A. Nelson . 2020. “Trophic Niche Size and Overlap Decreases With Increasing Ecosystem Productivity.” Oikos 129: 1303–1313.

[ece374215-bib-0060] Losos, J. B. 2008. “Phylogenetic Niche Conservatism, Phylogenetic Signal and the Relationship Between Phylogenetic Relatedness and Ecological Similarity Among Species.” Ecology Letters 11: 995–1003. 18673385 10.1111/j.1461-0248.2008.01229.x

[ece374215-bib-0061] Lu, M. , and W. Jetz . 2023. “Scale‐Sensitivity in the Measurement and Interpretation of Environmental Niches.” Trends in Ecology & Evolution 38: 554–567. 36803985 10.1016/j.tree.2023.01.003

[ece374215-bib-0062] Lüdecke, D. , M. S. Ben‐Shachar , I. Patil , P. Waggoner , and D. Makowski . 2021. “Performance: An R Package for Assessment, Comparison and Testing of Statistical Models.” Journal of Open Source Software 6: 3139.

[ece374215-bib-0063] Macarthur, R. , and R. Levins . 1967a. “The Limiting Similarity, Convergence, and Divergence of Coexisting Species.” American Naturalist 101: 377–385.

[ece374215-bib-0064] MacArthur, R. , and R. Levins . 1967b. “Divergence of Coexisting Species.” American Naturalist 101: 377–385.

[ece374215-bib-0065] Manlick, P. J. , and J. N. Pauli . 2020. “Human Disturbance Increases Trophic Niche Overlap in Terrestrial Carnivore Communities.” Proceedings of the National Academy of Sciences of the United States of America 117: 26842–26848. 33046630 10.1073/pnas.2012774117 PMC7604423

[ece374215-bib-0066] Mason, N. W. H. , F. de Bello , J. Dolez , and J. Leps . 2011. “Niche Overlap Reveals the Effects of Competition, Disturbance and Contrasting Assembly Processes in Experimental Grassland Communities.” Journal of Animal Ecology 99: 788–796.

[ece374215-bib-0067] Matthiopoulos, J. 2022. “Defining, Estimating, and Understanding the Fundamental Niches of Complex Animals in Heterogeneous Environments.” Ecological Monographs 92: e1545.

[ece374215-bib-0068] Mertes, K. , and W. Jetz . 2018. “Disentangling Scale Dependencies in Species Environmental Niches and Distributions.” Ecography (Copenhagen) 41: 1604–1615.

[ece374215-bib-0069] Münkemüller, T. , S. Lavergne , B. Bzeznik , et al. 2012. “How to Measure and Test Phylogenetic Signal.” Methods in Ecology and Evolution 3: 743–756.

[ece374215-bib-0070] Oksanen, J. , F. G. Blanchet , R. Kindt , et al. 2015. “vegan: Community Ecology Package.” http://cran.r‐project.org/package=vegan.

[ece374215-bib-0071] Ong, T. W. , B. B. Lin , A. Lucatero , et al. 2022. “Rarity Begets Rarity: Social and Environmental Drivers of Rare Organisms in Cities.” Ecological Applications 32: 1–19. 10.1002/eap.2708 PMC10078586 35810452

[ece374215-bib-0072] Pavoine, S. , M. B. Bonsall , A. Dupaix , U. Jacob , and C. Ricotta . 2017. “From Phylogenetic to Functional Originality: Guide Through Indices and New Developments.” Ecological Indicators 82: 196–205.

[ece374215-bib-0073] Pavoine, S. , and C. Ricotta . 2021. “On the Relationships Between Rarity, Uniqueness, Distinctiveness, Originality and Functional/Phylogenetic Diversity.” Biological Conservation 263: 109356.

[ece374215-bib-0074] Pearman, P. B. , A. Guisan , O. Broennimann , and C. F. Randin . 2008. “Niche Dynamics in Space and Time.” Trends in Ecology & Evolution 23: 149–158. 18289716 10.1016/j.tree.2007.11.005

[ece374215-bib-0075] Pebesma, E. , and R. Bivand . 2023. Spatial Data Science: With Applications in R. Chapman and Hall/CRC.

[ece374215-bib-0076] Pebesma, E. J. , and R. Bivand . 2005. “Classes and Methods for Spatial Data in R.” R News 5: 9–13.

[ece374215-bib-0077] Peterson, A. T. , and J. Soberón . 2012. “Species Distribution Modeling and Ecological Niche Modeling: Getting the Concepts Right.” Natureza & Conservação 10: 102–107.

[ece374215-bib-0078] Peterson, A. T. , J. Soberón , R. G. Pearson , et al. 2011. Ecological Niches and Geographic Distributions. *Monogr. Popul. Biol*. Princeton University Press.

[ece374215-bib-0079] Pianka, E. R. 1974. “Niche Overlap and Diffuse Competition.” Proceedings of the National Academy of Sciences of the United States of America 71: 2141–2145. 4525324 10.1073/pnas.71.5.2141 PMC388403

[ece374215-bib-0080] Pigot, A. L. , W. Jetz , C. Sheard , and J. A. Tobias . 2018. “The Macroecological Dynamics of Species Coexistence in Birds.” Nature Ecology & Evolution 2: 1112–1119. 29915343 10.1038/s41559-018-0572-9

[ece374215-bib-0081] Pinsky, M. L. , A. M. Eikeset , D. J. McCauley , J. L. Payne , and J. M. Sunday . 2019. “Greater Vulnerability to Warming of Marine Versus Terrestrial Ectotherms.” Nature 569: 108–111. 31019302 10.1038/s41586-019-1132-4

[ece374215-bib-0082] Qian, H. , and Y. Jin . 2016. “An Updated Megaphylogeny of Plants, a Tool for Generating Plant Phylogenies and an Analysis of Phylogenetic Community Structure.” Journal of Plant Ecology 9: 233–239.

[ece374215-bib-0083] R Core Team . 2023. R: A Language and Environment for Statistical Computing. R Foundation for Statistical Computing.

[ece374215-bib-0084] Rabosky, D. L. , J. Chang , P. O. Title , et al. 2018. “An Inverse Latitudinal Gradient in Speciation Rate for Marine Fishes.” Nature 559: 392–395. 29973726 10.1038/s41586-018-0273-1

[ece374215-bib-0085] Raes, N. , and H. Steege . 2007. “A Null‐Model for Significance Testing of Presence‐Only Species Distribution Models.” Ecography 30: 727–736.

[ece374215-bib-0086] Revell, L. J. 2012. “Phytools: An R Package for Phylogenetic Comparative Biology (And Other Things).” Methods in Ecology and Evolution 3: 217–223.

[ece374215-bib-0087] Ricklefs, R. E. 1987. “Community Diversity: Relative Roles of Local and Regional Processes.” Science (80‐) 235: 167–171. 10.1126/science.235.4785.167 17778629

[ece374215-bib-0088] Ricklefs, R. E. 2012. “Species Richness and Morphological Diversity of Passerine Birds.” Proceedings of the National Academy of Sciences of the United States of America 109: 14482–14487. 22908271 10.1073/pnas.1212079109 PMC3437851

[ece374215-bib-0089] Rödder, D. , and J. O. Engler . 2011. “Quantitative Metrics of Overlaps in Grinnellian Niches: Advances and Possible Drawbacks.” Global Ecology and Biogeography 20: 915–927.

[ece374215-bib-0090] Slatyer, R. A. , M. Hirst , and J. P. Sexton . 2013. “Niche Breadth Predicts Geographical Range Size: A General Ecological Pattern.” Ecology Letters 16: 1104–1114. 23773417 10.1111/ele.12140

[ece374215-bib-0091] Soberón, J. 2007. “Grinnellian and Eltonian Niches and Geographic Distributions of Species.” Ecology Letters 10: 1115–1123. 17850335 10.1111/j.1461-0248.2007.01107.x

[ece374215-bib-0092] Steele, J. H. 1991. “Can Ecological Theory Cross the Land‐Sea Boundary?” Journal of Theoretical Biology 153: 425–436.

[ece374215-bib-0093] Stein, A. , K. Gerstner , and H. Kreft . 2014. “Environmental Heterogeneity as a Universal Driver of Species Richness Across Taxa, Biomes and Spatial Scales.” Ecology Letters 17: 866–880. 24751205 10.1111/ele.12277

[ece374215-bib-0094] Stein, R. W. , C. G. Mull , T. S. Kuhn , et al. 2018. “Global Priorities for Conserving the Evolutionary History of Sharks, Rays and Chimaeras.” Nature Ecology & Evolution 2: 288–298. 29348644 10.1038/s41559-017-0448-4

[ece374215-bib-0095] Suraci, J. P. , J. A. Smith , S. Chamaillé‐Jammes , et al. 2022. “Beyond Spatial Overlap: Harnessing New Technologies to Resolve the Complexities of Predator–Prey Interactions.” Oikos 2022: 1–15.

[ece374215-bib-0096] Swanson, H. K. , M. Lysy , M. Power , A. D. Statsko , J. D. Johnson , and J. D. Reist . 2015. “A New Probabilistic Method for Quantifying n‐Dimensional Ecological Niches and Niche Overlap.” Ecology 96: 318–324. 26240852 10.1890/14-0235.1

[ece374215-bib-0097] Tonini, J. F. R. , K. H. Beard , R. B. Ferreira , W. Jetz , and R. A. Pyron . 2016. “Fully‐Sampled Phylogenies of Squamates Reveal Evolutionary Patterns in Threat Status.” Biological Conservation 204: 23–31.

[ece374215-bib-0098] Trisos, C. H. , C. Merow , and A. L. Pigot . 2020. “The Projected Timing of Abrupt Ecological Disruption From Climate Change.” Nature 580: 496–501. 32322063 10.1038/s41586-020-2189-9

[ece374215-bib-0099] Tucker, C. M. , M. W. Cadotte , S. B. Carvalho , et al. 2017. “A Guide to Phylogenetic Metrics for Conservation, Community Ecology and Macroecology.” Biological Reviews 92: 698–715. 26785932 10.1111/brv.12252 PMC5096690

[ece374215-bib-0100] Tyberghein, L. , H. Verbruggen , K. Pauly , C. Troupin , F. Mineur , and O. De Clerck . 2012. “Bio‐ORACLE: A Global Environmental Dataset for Marine Species Distribution Modelling.” Global Ecology and Biogeography 21: 272–281.

[ece374215-bib-0101] Tyler, E. H. M. , P. J. Somerfield , E. Vanden Berghe , et al. 2012. “Extensive Gaps and Biases in Our Knowledge of a Well‐Known Fauna Traits Macroecology.” Global Ecology and Biogeography 21: 922–934.

[ece374215-bib-0102] Upham, N. S. , J. A. Esselstyn , and W. Jetz . 2019. “Inferring the Mammal Tree: Species‐Level Sets of Phylogenies for Questions in Ecology, Evolution, and Conservation.” PLoS Biology 17: e3000494. 31800571 10.1371/journal.pbio.3000494 PMC6892540

[ece374215-bib-0103] Vellend, M. 2010. “Conceptual Synthesis in Community Ecology.” Quarterly Review of Biology 85: 183–206. 20565040 10.1086/652373

[ece374215-bib-0104] Vellend, M. 2016. The Theory of Ecological Communities. Princeton University Press.

[ece374215-bib-0105] Villalobos, F. , R. Dobrovolski , D. B. Provete , and S. F. Gouveia . 2013. “Is Rich and Rare the Common Share? Describing Biodiversity Patterns to Inform Conservation Practices for South American Anurans.” PLoS One 8: e56073. 23409124 10.1371/journal.pone.0056073 PMC3567056

[ece374215-bib-0106] Violle, C. , M. L. Navas , D. Vile , et al. 2007. “Let the Concept of Trait Be Functional!” Oikos 116: 882–892.

[ece374215-bib-0107] Violle, C. , P. B. Reich , S. W. Pacala , B. J. Enquist , and J. Kattge . 2014. “The Emergence and Promise of Functional Biogeography.” Proceedings of the National Academy of Sciences of the United States of America 111: 13690–13696. 25225414 10.1073/pnas.1415442111 PMC4183284

[ece374215-bib-0108] Violle, C. , W. Thuiller , N. Mouquet , et al. 2017. “Functional Rarity: The Ecology of Outliers.” Trends in Ecology & Evolution 32: 356–367. 28389103 10.1016/j.tree.2017.02.002 PMC5489079

[ece374215-bib-0109] Waldock, C. , R. D. Stuart‐Smith , G. J. Edgar , T. J. Bird , and A. E. Bates . 2019. “The Shape of Abundance Distributions Across Temperature Gradients in Reef Fishes.” Ecology Letters 22: 685–696. 30740843 10.1111/ele.13222 PMC6850591

[ece374215-bib-0110] Warren, D. L. , M. Cardillo , D. F. Rosauer , and D. I. Bolnick . 2014. “Mistaking Geography for Biology: Inferring Processes From Species Distributions.” Trends in Ecology & Evolution 29: 572–580. 25172405 10.1016/j.tree.2014.08.003

[ece374215-bib-0111] Webb, C. O. , D. D. Ackerly , M. A. McPeek , and M. J. Donoghue . 2002. “Phylogenies and Community Ecology.” Annual Review of Ecology and Systematics 33: 475–505.

[ece374215-bib-0112] Weiher, E. , and P. A. Keddy . 1995. “Assembly Rules, Null Models, and Trait Dispersion: New Questions From Old Patterns.” Oikos 74: 159–164.

[ece374215-bib-0113] Wickham, H. 2016. ggplot2: Elegant Graphics for Data Analysis. Springer‐Verlag.

[ece374215-bib-0114] Wiens, J. J. 2011. “The Niche, Biogeography and Species Interactions.” Philosophical Transactions of the Royal Society, B: Biological Sciences 366: 2336–2350. 10.1098/rstb.2011.0059 PMC3130432 21768150

[ece374215-bib-0115] Wiens, J. J. , D. D. Ackerly , A. P. Allen , et al. 2010. “Niche Conservatism as an Emerging Principle in Ecology and Conservation Biology.” Ecology Letters 13: 1310–1324. 20649638 10.1111/j.1461-0248.2010.01515.x

[ece374215-bib-0116] Worm, B. , and D. P. Tittensor . 2018. A Theory of Global Biodiversity (MPB‐60). Princeton University Press.

[ece374215-bib-0117] Zanne, A. E. , D. C. Tank , W. K. Cornwell , et al. 2014. “Three Keys to the Radiation of Angiosperms Into Freezing Environments.” Nature 506: 89–92. 24362564 10.1038/nature12872

[ece374215-bib-0118] Zhao, M. , F. A. Heinsch , R. R. Nemani , and S. W. Running . 2005. “Improvements of the MODIS Terrestrial Gross and Net Primary Production Global Data Set.” Remote Sensing of Environment 95: 164–176.

