# Supplementary material for: Global Patterns and Drivers of Climatic Niche Originality and Overlap: Disentangling Ecological From Methodological Signals
Source: Ecol Evol. 2026 Sep 3;16(9):e74215. doi: 10.1002/ece3.74215 (PMC13539904; doi:10.1002/ece3.74215)
Supplement: Supplementary file 1 [file ECE3-16-e74215-s001.docx]

**Supplementary material -** Global patterns and drivers of climatic niche originality and overlap: disentangling ecological from methodological signals

Mathieu Chevalier, Clément Violet and Aurélien Boyé

**Supplementary text 1 - SEM diagnostics**

Visual inspection of the diagnostic plots did not reveal any major violations of model assumptions or obvious lack of fit. Residuals were approximately normally distributed, although Q–Q plots indicated mild to moderate departures in the tails only for some component models. Residuals-versus-fitted and scale-location plots suggested modest heteroscedasticity and systematic residual trends in most component models, indicating that the linear specification does not fully capture all of the structure in the data and may reflect unmodelled non-linear relationships and/or omitted predictors. Nevertheless, multicollinearity was low across all component models (maximum VIF = 3.86), and the observed departures were not considered sufficiently severe to invalidate inference. We therefore retained a common linear model structure throughout the SEM analyses, as introducing quadratic or other non-linear terms would have substantially increased model complexity, reduced the interpretability of path coefficients, and compromised direct comparisons of structural relationships among taxonomic groups. Representative diagnostic plots are presented in Supplementary Fig. S1-S8, while summary diagnostic statistics for all component models are provided in Supplementary Table S1.

| Table S1. Summary of model fit and diagnostic statistics for component models of the piecewise structural equation models across taxonomic groups. Marginal and conditional R² values quantify variance explained by fixed effects and by fixed plus random effects, respectively. Residual skewness and kurtosis, Breusch–Pagan (BP) test statistics, BP p-values, and maximum variance inflation factors (VIF) are provided for diagnostic assessment. Given the exceptionally large sample sizes of several datasets, Breusch–Pagan tests had high statistical power and detected statistically significant departures from homoscedasticity even when deviations were modest. Therefore, evidence for heteroscedasticity was interpreted based on the magnitude of residual patterns and visual inspection of diagnostic plots rather than statistical significance alone. | | | | | | | | | |
| --- | --- | --- | --- | --- | --- | --- | --- | --- | --- |
| Taxonomic group | Response | N | Marginal r² | Conditional r² | Skewness | Kurtosis | Bp stat | Bp p-value | Max vif |
| Amphibians | Niche overlap | 28925 | 0.38 | 0.95 | 0.14 | 3.27 | 3568.37 | <0.001 | 1.09 |
|  | Range overlap | 28925 | 0.54 | 0.95 | 0.47 | 3.98 | 4252.57 | <0.001 | 1.80 |
|  | Max niche breadth | 28925 | 0.06 | 0.97 | 0.39 | 4.87 | 1852.48 | <0.001 | 1.03 |
| Birds | Niche overlap | 1663258 | 0.36 | 0.95 | 0.12 | 3.19 | 224099.62 | <0.001 | 1.15 |
|  | Range overlap | 1663258 | 0.51 | 0.95 | 0.76 | 5.40 | 277749.79 | <0.001 | 1.75 |
|  | Max niche breadth | 1663258 | 0.12 | 0.96 | 0.72 | 5.78 | 85710.07 | <0.001 | 1.04 |
| Condrichthyes | Niche overlap | 27270 | 0.39 | 0.95 | 0.07 | 3.01 | 1044.93 | <0.001 | 1.17 |
|  | Range overlap | 27270 | 0.28 | 0.93 | 1.63 | 8.25 | 2255.65 | <0.001 | 1.89 |
|  | Max niche breadth | 27270 | 0.12 | 0.94 | -0.03 | 3.39 | 702.61 | <0.001 | 1.01 |
| Plants | Niche overlap | 14193 | 0.47 | 0.97 | 0.14 | 7.61 | 1795.01 | <0.001 | 3.85 |
|  | Range overlap | 14193 | 0.71 | 0.97 | -0.36 | 3.43 | 587.71 | <0.001 | 3.86 |
|  | Max niche breadth | 14193 | 0.61 | 0.97 | 0.60 | 7.80 | 1508.58 | <0.001 | 1.10 |
| Terrestrial mammals | Niche overlap | 235876 | 0.35 | 0.95 | 0.01 | 3.42 | 35719.94 | <0.001 | 1.09 |
|  | Range overlap | 235876 | 0.55 | 0.95 | 0.65 | 5.33 | 45860.10 | <0.001 | 1.65 |
|  | Max niche breadth | 235876 | 0.07 | 0.96 | 0.55 | 5.54 | 16299.56 | <0.001 | 1.01 |
| Marine fish | Niche overlap | 196645 | 0.59 | 0.96 | 0.20 | 3.47 | 7296.30 | <0.001 | 1.35 |
|  | Range overlap | 196645 | 0.55 | 0.96 | 0.27 | 3.80 | 3650.64 | <0.001 | 2.60 |
|  | Max niche breadth | 196645 | 0.16 | 0.95 | 0.60 | 4.60 | 12587.86 | <0.001 | 1.01 |
| Freshwater fish | Niche overlap | 43633 | 0.43 | 0.95 | 0.18 | 3.37 | 5929.16 | <0.001 | 1.17 |
|  | Range overlap | 43633 | 0.45 | 0.94 | 1.00 | 6.10 | 7895.95 | <0.001 | 1.89 |
|  | Max niche breadth | 43633 | 0.08 | 0.97 | 0.94 | 5.64 | 898.85 | <0.001 | 1.09 |
| Reptiles | Niche overlap | 101477 | 0.38 | 0.95 | 0.26 | 3.36 | 13969.52 | <0.001 | 1.43 |
|  | Range overlap | 101477 | 0.56 | 0.95 | 0.42 | 4.81 | 14798.90 | <0.001 | 2.03 |
|  | Max niche breadth | 101477 | 0.21 | 0.97 | 0.79 | 5.32 | 6194.90 | <0.001 | 1.06 |

**Fig. S1.** Residual diagnostic plots for the piecewise structural equation models fitted on Amphibians. Each row corresponds to one component model of the SEM, representing (from top to bottom) range overlap, niche overlap, and maximum niche breadth (log transform). Columns show (from left to right): residuals versus fitted values, normal Q–Q plots, scale-location plots, and residuals versus the main predictor. Red lines represent locally estimated smoothing trends, and dashed horizontal lines indicate zero residuals. For computational efficiency, datasets containing more than 10,000 observations were randomly subsampled for visualization only; model fitting was performed using the complete datasets. These diagnostics were used to assess residual structure, normality, variance homogeneity, and potential model misspecification across SEM component models.

**Fig. S2.** Residual diagnostic plots for the piecewise structural equation models fitted on Birds. Each row corresponds to one component model of the SEM, representing (from top to bottom) range overlap, niche overlap, and maximum niche breadth (log transform). Columns show (from left to right): residuals versus fitted values, normal Q–Q plots, scale-location plots, and residuals versus the main predictor. Red lines represent locally estimated smoothing trends, and dashed horizontal lines indicate zero residuals. For computational efficiency, datasets containing more than 10,000 observations were randomly subsampled for visualization only; model fitting was performed using the complete datasets. These diagnostics were used to assess residual structure, normality, variance homogeneity, and potential model misspecification across SEM component models.

**Fig. S3.** Residual diagnostic plots for the piecewise structural equation models fitted on Condrichthyes. Each row corresponds to one component model of the SEM, representing (from top to bottom) range overlap, niche overlap, and maximum niche breadth (log transform). Columns show (from left to right): residuals versus fitted values, normal Q–Q plots, scale-location plots, and residuals versus the main predictor. Red lines represent locally estimated smoothing trends, and dashed horizontal lines indicate zero residuals. For computational efficiency, datasets containing more than 10,000 observations were randomly subsampled for visualization only; model fitting was performed using the complete datasets. These diagnostics were used to assess residual structure, normality, variance homogeneity, and potential model misspecification across SEM component models.

**Fig. S4.** Residual diagnostic plots for the piecewise structural equation models fitted on Freshwater Fish. Each row corresponds to one component model of the SEM, representing (from top to bottom) range overlap, niche overlap, and maximum niche breadth (log transform). Columns show (from left to right): residuals versus fitted values, normal Q–Q plots, scale-location plots, and residuals versus the main predictor. Red lines represent locally estimated smoothing trends, and dashed horizontal lines indicate zero residuals. For computational efficiency, datasets containing more than 10,000 observations were randomly subsampled for visualization only; model fitting was performed using the complete datasets. These diagnostics were used to assess residual structure, normality, variance homogeneity, and potential model misspecification across SEM component models.

**Fig. S5.** Residual diagnostic plots for the piecewise structural equation models fitted on Terrestrial Mammals. Each row corresponds to one component model of the SEM, representing (from top to bottom) range overlap, niche overlap, and maximum niche breadth (log transform). Columns show (from left to right): residuals versus fitted values, normal Q–Q plots, scale-location plots, and residuals versus the main predictor. Red lines represent locally estimated smoothing trends, and dashed horizontal lines indicate zero residuals. For computational efficiency, datasets containing more than 10,000 observations were randomly subsampled for visualization only; model fitting was performed using the complete datasets. These diagnostics were used to assess residual structure, normality, variance homogeneity, and potential model misspecification across SEM component models.

**Fig. S6.** Residual diagnostic plots for the piecewise structural equation models fitted on Marine Fish. Each row corresponds to one component model of the SEM, representing (from top to bottom) range overlap, niche overlap, and maximum niche breadth (log transform). Columns show (from left to right): residuals versus fitted values, normal Q–Q plots, scale-location plots, and residuals versus the main predictor. Red lines represent locally estimated smoothing trends, and dashed horizontal lines indicate zero residuals. For computational efficiency, datasets containing more than 10,000 observations were randomly subsampled for visualization only; model fitting was performed using the complete datasets. These diagnostics were used to assess residual structure, normality, variance homogeneity, and potential model misspecification across SEM component models.

**Fig. S7.** Residual diagnostic plots for the piecewise structural equation models fitted on Plants. Each row corresponds to one component model of the SEM, representing (from top to bottom) range overlap, niche overlap, and maximum niche breadth (log transform). Columns show (from left to right): residuals versus fitted values, normal Q–Q plots, scale-location plots, and residuals versus the main predictor. Red lines represent locally estimated smoothing trends, and dashed horizontal lines indicate zero residuals. For computational efficiency, datasets containing more than 10,000 observations were randomly subsampled for visualization only; model fitting was performed using the complete datasets. These diagnostics were used to assess residual structure, normality, variance homogeneity, and potential model misspecification across SEM component models.

**Fig. S8.** Residual diagnostic plots for the piecewise structural equation models fitted on Reptiles. Each row corresponds to one component model of the SEM, representing (from top to bottom) range overlap, niche overlap, and maximum niche breadth (log transform). Columns show (from left to right): residuals versus fitted values, normal Q–Q plots, scale-location plots, and residuals versus the main predictor. Red lines represent locally estimated smoothing trends, and dashed horizontal lines indicate zero residuals. For computational efficiency, datasets containing more than 10,000 observations were randomly subsampled for visualization only; model fitting was performed using the complete datasets. These diagnostics were used to assess residual structure, normality, variance homogeneity, and potential model misspecification across SEM component models.

| Table S2. Summary statistics for different measures present in the BioOverlap database obtained from species-wise estimates of 3D niche hypervolumes and 2D range polygons. | | | | | | | |
| --- | --- | --- | --- | --- | --- | --- | --- |
| Realm | Type of overlap | Summary statistic | Volume/Area | Centroid distance | Edge-to-edge distance | Jaccard | Sorensen |
| Terrestrial | Niche | Min | 0.05 | 0.00 | 0.00 | 0.00 | 0.00 |
|  |  | Max | 2301.85 | 13.46 | 20002.71 | 0.96 | 0.98 |
|  |  | Mean | 61.61 | 3.72 | 6691.43 | 0.10 | 0.15 |
|  |  | Median | 21.44 | 3.35 | 5890.14 | 0.04 | 0.07 |
|  |  | Sd | 111.87 | 2.19 | 5344.66 | 0.14 | 0.19 |
|  | Range | Min | 0.05 | 0.00 | 0.00 | 0.00 | 0.00 |
|  |  | Max | 30733.91 | 20003.86 | 20002.71 | 1.00 | 1.00 |
|  |  | Mean | 389.70 | 9323.52 | 6691.43 | 0.01 | 0.02 |
|  |  | Median | 157.58 | 9375.06 | 5890.14 | 0.00 | 0.00 |
|  |  | Sd | 965.93 | 4962.30 | 5344.66 | 0.07 | 0.09 |
| Marine | Niche | Min | 0.38 | 0.00 | 0.00 | 0.00 | 0.00 |
|  |  | Max | 2069.84 | 11.52 | 19980.27 | 0.96 | 0.98 |
|  |  | Mean | 137.88 | 2.11 | 4033.63 | 0.29 | 0.41 |
|  |  | Median | 88.49 | 1.92 | 1126.94 | 0.26 | 0.41 |
|  |  | Sd | 161.60 | 1.32 | 5240.61 | 0.21 | 0.25 |
|  | Range | Min | 20.09 | 0.00 | 0.00 | 0.00 | 0.00 |
|  |  | Max | 22354.59 | 20000.63 | 19980.27 | 1.00 | 1.00 |
|  |  | Mean | 718.25 | 8560.98 | 4033.63 | 0.08 | 0.11 |
|  |  | Median | 295.35 | 7635.02 | 1126.94 | 0.00 | 0.00 |
|  |  | Sd | 1454.51 | 5610.38 | 5240.61 | 0.17 | 0.22 |

**Figure S9.** Boxplots highlighting niche overlap between species that are found in sympatry or in allopatry for the different taxonomic groups present in the BioOverlap database.

**Figure S10.** Boxplots highlighting niche overlap between species within the same taxonomic group (Within) or in different taxonomic groups (Between).

**Figure S11.** Sensitivity analysis related to the drivers of niche originality when measured using the mean distance to the ten closest neighbors in the environmental space. For further details, see Figure 3 in the main text.
